# A Review on Low-Dimensional Nanoarchitectonics for Neurochemical Sensing and Modulation in Responsive Neurological Outcomes

**DOI:** 10.3390/biom15101405

**Published:** 2025-10-02

**Authors:** Mohammad Tabish, Iram Malik, Ali Akhtar, Mohd Afzal

**Affiliations:** 1 Department of Pharmacology, College of Medicine, Shaqra University, Shaqra 11961, Saudi Arabia; 2 Department of Electrical Engineering, College of Engineering, Prince Sattam bin Abdulaziz University, Al-Kharj 11942, Saudi Arabia; i.malik@psau.edu.sa; 3 King Salman Center for Disability Research, Riyadh 11614, Saudi Arabia; 4 Department of Pharmacognosy, College of Pharmacy, King Saud University, Riyadh 11451, Saudi Arabia; aakhtar@ksu.edu.sa; 5 Department of Chemistry, College of Science, King Saud University, Riyadh 11451, Saudi Arabia

**Keywords:** low-dimensional nanohybrids, neuromodulation, artificial intelligence, brain–computer interfaces, closed-loop systems, precision neurotechnology

## Abstract

Low-Dimensional Nanohybrids (LDNHs) have emerged as potent multifunctional platforms for neurosensing and neuromodulation, providing elevated spatial-temporal precision, versatility, and biocompatibility. This review examines the intersection of LDNHs with artificial intelligence, brain–computer interfaces (BCIs), and closed-loop neurotechnologies, highlighting their transformative potential in personalized neuro-nano-medicine. Utilizing stimuli-responsive characteristics, optical, thermal, magnetic, and electrochemical LDNHs provide real-time feedback-controlled manipulation of brain circuits. Their pliable and adaptable structures surpass the constraints of inflexible bioelectronics, improving the neuronal interface and reducing tissue damage. We also examined their use in less invasive neurological diagnostics, targeted therapy, and adaptive intervention systems. This review delineates recent breakthroughs, integration methodologies, and fundamental mechanisms, while addressing significant challenges such as long-term biocompatibility, deep-tissue accessibility, and scalable manufacturing. A strategic plan is provided to direct future research toward clinical use. Ultimately, LDNHs signify a transformative advancement in intelligent, tailored, and closed-loop neurotechnologies, integrating materials science, neurology, and artificial intelligence to facilitate the next era of precision medicine.

## 1. Introduction

Neurological disorders represent a major and escalating global health burden, afflicting hundreds of millions of individuals and accounting for a significant proportion of disability-adjusted life years (DALYs) [1]. Conditions such as Parkinson’s disease, Alzheimer’s disease, depression, epilepsy, and traumatic brain injuries are not only devastating at the personal level but also place immense strain on healthcare systems worldwide [2]. Despite extensive progress in neuroscience and neuropharmacology, early diagnosis, real-time monitoring, and precision interventions in neurodegenerative and neuropsychiatric diseases remain severely limited [3]. One of the critical barriers is the inability to monitor neurochemical changes in the brain with sufficient spatiotemporal resolution and molecular specificity. Neurochemicals such as dopamine, glutamate, serotonin, γ-aminobutyric acid (GABA), and acetylcholine orchestrate the intricate electrochemical signaling networks underlying cognition, mood, motor function, and memory [4]. Even subtle dysregulations in their concentrations can lead to severe and often irreversible neuropathological changes [5]. However, the dynamic and low-abundance nature of these molecules, combined with the complex biochemical microenvironment of the brain, presents formidable challenges in their analysis. Traditional neurochemical sensing modalities, including microdialysis, magnetic resonance spectroscopy, and enzyme-linked assays, are hindered by a lack of real-time capabilities, low sensitivity, and invasiveness, which compromise both data quality and long-term applicability [6,7]. Likewise, conventional neuromodulation tools, such as deep brain stimulation (DBS), pharmacological agents, and optogenetics, often lack chemical specificity, suffer from systemic side effects, or require genetic manipulation, which limits their translational potential. In recent years, the convergence of nanotechnology and neuroscience has ushered in transformative possibilities for neurochemical interfacing [8,9,10]. In particular, low-dimensional nanomaterials, defined as materials with confined dimensions at the nanoscale in one (1D), two (2D), or all three (0D) spatial directions, have emerged as promising candidates for revolutionizing brain–device interfaces. These LDNHs, formed by the rational integration of distinct nanomaterials such as quantum dots (QDs) [11,12,13,14,15,16,17,18,19,20], carbon nanotubes (CNTs) [21,22,23,24], graphene [25,26,27,28,29,30], MXenes [31,32,33,34,35], and black phosphorus (BP) [36,37], offer an unprecedented combination of electrical conductivity, high surface-to-volume ratio, chemical tunability, flexibility, and biocompatibility [38,39,40]. LDNHs transcend the limitations of individual nanomaterials by synergistically combining their functionalities via hybridization strategies. For instance, the integration of 0D QDs with 2D graphene sheets enhances the charge transfer efficiency and photoluminescence for ultrasensitive neurochemical sensing, while CNT–MXene composites exhibit exceptional electrochemical performance and mechanical compliance for implantable neural electrodes [41]. These hybrid systems can be precisely engineered at the nanoscale to achieve tailored properties for detecting specific neurotransmitters or modulating targeted brain regions with high fidelity [41,42]. Furthermore, advances in surface functionalization and biointerface engineering, such as PEGylation, peptide anchoring, and bioinspired coatings, have significantly improved the stability, selectivity, and in vivo compatibility of LDNHs [43].

A particularly compelling application of LDNHs is in real-time in vivo neurochemical sensing. Electrochemical sensors based on LDNHs can detect redox-active neurotransmitters with high temporal resolution, enabling dynamic mapping of neuronal activity in response to physiological or pharmacological stimuli [44]. Optical sensors incorporating fluorescent carbon dots or SERS-active nanohybrids allow for non-invasive imaging of neurochemical events with subcellular spatial precision. The integration of these sensors into flexible, minimally invasive platforms, such as neural probes, wearables, or wireless implants, further paves the way for continuous monitoring in both clinical and research settings. Importantly, LDNHs can also function as active components in neuromodulatory systems [45]. Electrically conductive nanohybrids facilitate localized brain stimulation with reduced impedance and enhanced charge injection, while photothermal or magnetothermal nanohybrids enable wireless, stimuli-responsive neuromodulation [46]. These capabilities open the door to closed-loop systems that not only sense but also modulate brain activity in real time, forming the basis of precision neurotherapeutics. Beyond their immediate biomedical applications, LDNHs also offer a versatile platform for fundamental neuroscience research. Their ability to interface with neurons, glial cells, and the extracellular matrix at the nanoscale allows for the exploration of brain dynamics with an unprecedented resolution [47]. LDNHs are now being explored in emerging domains such as brain–computer interfaces (BCIs), neural organoids, and lab-on-a-chip systems that recapitulate complex neural circuits [48]. Their tunable physicochemical properties enable the design of multifunctional probes capable of integrating electrical, optical, and biochemical modalities into a single miniaturized platform [49]. The convergence of these capabilities with artificial intelligence (AI) and big data analytics has the potential to accelerate discovery, decode brain disorders, and develop next-generation neurotechnologies [50]. Despite their promise, the translation of LDNHs from bench to bedside remains in its infancy. Key challenges include long-term biocompatibility, potential toxicity, immunogenicity, and difficulty in integrating nanoscale devices with the soft, dynamic tissue of the brain [51]. Regulatory and ethical considerations, particularly concerning neural privacy, safety, and data ownership, further complicate the deployment of LDNH-based neurodevices in human populations [52]. A comprehensive understanding of the interaction between nanomaterials and the central nervous system (CNS), especially in chronic implantation scenarios, is urgently required [53]. Moreover, scalable and reproducible fabrication methods for clinical-grade nanohybrids must be established to ensure quality control, consistency, and affordability [53].

This review provides a critical and forward-looking synthesis of recent advances, emerging opportunities, and translational challenges in the field of low-dimensional nanohybrids for neurochemical sensing and neuromodulation. We begin by outlining the neurochemical architecture of the brain and the functional relevance of key signaling molecules in health and in disease. We then explore the design principles and material innovations that underpin LDNH-based sensing and modulation platforms, highlighting the major classes of hybrid nanomaterials and their integration strategies. This review further discusses the state-of-the-art applications of LDNHs in real-time biosensing, neural stimulation, and closed-loop feedback systems, emphasizing their clinical potential and limitations. We also examine the critical role of biointerface engineering in achieving stable, non-immunogenic integration with neural tissue. Finally, we present an outlook on future directions in the field, including the fusion of LDNHs with wireless electronics, AI-enabled diagnostics, and personalized neurotherapeutics. Relevant studies were identified and screened following the PRISMA 2020 guidelines, with the overall selection process summarized in the flow diagram (Supporting Information; Figure S1).

By bridging materials science, neuroscience, and biomedical engineering, LDNHs have the potential to redefine the neurotechnology paradigm. They offer not only an enhanced understanding of the molecular underpinnings of brain function but also a tangible path toward responsive, adaptive, and patient-specific interventions for treating neurological disorders. As this interdisciplinary field continues to evolve, close collaboration between nanotechnologists, neuroengineers, clinicians, and ethicists will be essential to ensure that these powerful technologies are developed safely, responsibly, and equitably for the benefit of society.

## 2. Neurochemicals and Their Modulation Challenges

Neurochemical signaling is fundamental to brain function and governs essential physiological and behavioral processes, such as memory, mood, attention, motor control, and circadian rhythms. Neurotransmitters and neuromodulators—ranging from amino acids like glutamate and GABA to monoamines like dopamine and serotonin—enable communication between neurons and glial cells across vast neural networks [54,55,56]. Precise spatial and temporal regulation of these chemicals is crucial for maintaining brain homeostasis [57]. Even minor deviations in their concentrations or signaling dynamics can lead to debilitating neurological or psychiatric conditions, including Alzheimer’s disease, Parkinson’s disease, major depressive disorder, epilepsy, schizophrenia, and chronic pain syndromes [58]. Despite their importance, accurate, real-time detection and modulation of these neurochemicals in vivo remains a formidable challenge due to their low concentrations, rapid turnover, regional specificity, and protective barriers of the central nervous system (CNS), most notably the blood–brain barrier (BBB) [59,60,61].

**Figure 1:**
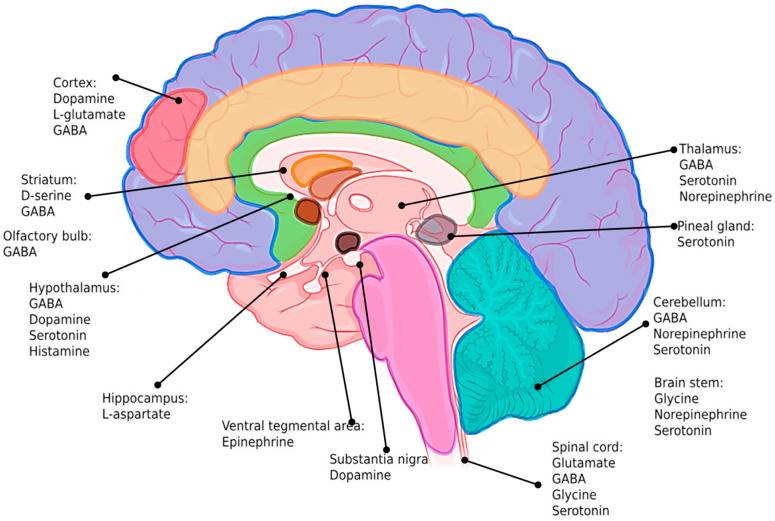
Regional distribution of major neurochemicals in the human brain. Different brain regions exhibit specific neurochemical environments. For instance, dopamine is predominant in the substantia nigra and cortex, GABA is widespread in inhibitory pathways throughout the cortex, cerebellum, and olfactory bulb, and serotonin is concentrated in the thalamus, pineal gland, and brainstem. This spatial heterogeneity underscores the need for region-specific sensing and modulation strategies.

Neurotransmitters are distributed heterogeneously across various brain regions, each exhibiting distinct neurochemical profiles tailored to their specialized functions [62]. As illustrated in Figure 1, different brain regions are characterized by dominant or co-localized neurochemical signatures. For example, the cerebral cortex, which is responsible for higher-order cognitive functions, is enriched in dopamine, glutamate, and GABA [63]. The striatum, a key structure involved in motor control and reward processing, contains high concentrations of D-serine and GABA [56]. The hippocampus, which is essential for learning and memory, is rich in L-aspartate, while the olfactory bulb and hypothalamus express abundant GABA, with the latter also containing dopamine, serotonin, and histamine [64]. Monoaminergic neurotransmitters, such as serotonin, norepinephrine, and epinephrine, are prominently distributed across the thalamus, brainstem, and spinal cord, reflecting their roles in sensory integration, mood regulation, autonomic control, and pain processing [65]. This complex neurochemical topography presents a major challenge for both diagnosis and treatment. Traditional techniques, such as microdialysis and high-performance liquid chromatography (HPLC), allow for chemical quantification but lack the temporal resolution and invasiveness required for dynamic, real-time analysis [66]. Furthermore, these methods often fail to capture transient fluctuations or localized gradients of neurotransmitters within functional neural circuits. The temporal dynamics of synaptic signaling occur on a millisecond scale, and spatial gradients can span micron scales that are difficult to probe using conventional macroscale sensors.

Additionally, neuromodulation strategies currently employed in clinical settings are largely electrical, relying on non-specific stimulation modalities, such as deep brain stimulation (DBS) [67]. While DBS has shown efficacy in disorders like Parkinson’s disease and dystonia, it lacks chemical specificity, affecting both pathological and healthy tissues [68]. Similarly, pharmacological approaches suffer from poor BBB permeability, off-target effects, rapid systemic clearance, and lack of tunability once administered. Optogenetics has introduced more precision by targeting specific neuron types; however, its reliance on genetic manipulation limits its immediate translational potential, especially in human patients [68]. Another layer of complexity arises from the dynamic interplay among neurotransmitters. Excitatory and inhibitory signals must be tightly balanced to maintain functional stability. Glutamate, the principal excitatory neurotransmitter, plays a vital role in synaptic plasticity and memory but can lead to excitotoxicity if dysregulated [69]. Conversely, GABA, the major inhibitory neurotransmitter, helps suppress overexcitation but is finely regulated by transporters and enzymatic degradation. Monoamines, such as dopamine, serotonin, and norepinephrine, often act as modulators rather than direct excitatory or inhibitory transmitters, adjusting the gain of neural circuits and influencing everything from mood to arousal. Their dysregulation has been implicated in a spectrum of disorders, including depression, ADHD, and addiction. Understanding and intervening in these networks requires tools capable of capturing multi-analyte interactions in real time, ideally in awake and behaving subjects. Moreover, neurotransmitter activity is often context- and state-dependent, varying with circadian rhythm, hormonal milieu, stress, and behavior [70]. Therefore, the design of next-generation neurochemical interfaces must consider not only analyte selectivity and sensitivity but also biological compatibility, real-time adaptability, and long-term integration with neural tissue. Biofouling, immune responses, and glial scarring can impair signal fidelity and render devices ineffective. Therefore, implantable sensors and modulators must exhibit mechanical softness, chemical inertness, and bioactive coatings to sustain their function in the highly sensitive environment of the brain. In this context, the advent of LDNHs offers an exciting opportunity to transcend the limitations of conventional neurochemical tools [71]. Their high surface area, exceptional electrical and optical properties, and tunable functionalization enable the design of multifunctional platforms for the highly sensitive, selective, and minimally invasive detection of neurochemicals. When engineered into electrodes, transistors, or fluorescent probes, LDNHs can bridge the gap between nanoscale molecular interactions and macroscale neurophysiological phenomena. Furthermore, their integration into flexible substrates and wireless systems opens new avenues for developing closed-loop, patient-specific neuromodulation technologies [72,73,74]. To fully realize this potential, however, a deeper understanding of the neurochemical landscape and its modulation challenges is required [75]. This includes not only mapping neurochemical circuits but also engineering LDNH-based tools capable of interacting with these circuits in a biocompatible, high-resolution, and dynamic manner. In the following sections, we explore the material platforms, sensing architectures, and therapeutic strategies enabled by LDNHs and how they can be tailored to meet the demands of next-generation neurotechnologies.

**Figure 2:**
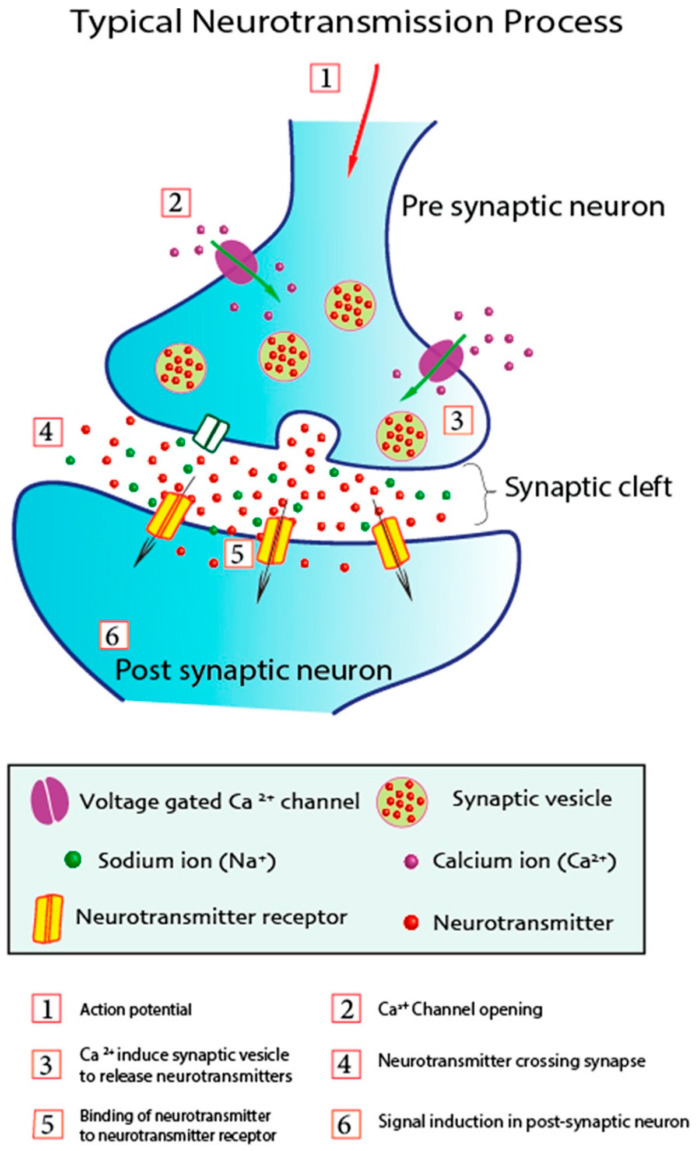
Schematic depiction of neurotransmission between presynaptic and postsynaptic neurons. Reproduced with permission from reference [77]. Copyright © 2020, American Chemical Society.

Neurotransmission primarily relies on the quantity of neurotransmitters, the pace of their release, the interaction between presynaptic neurotransmitter and postsynaptic receptor, and their subsequent activity [76]. Figure 2 illustrates the mechanism of neurotransmission between presynaptic and postsynaptic neurons. Upon the arrival of the action potential to the axonal terminal of the presynaptic neuron, it induces the opening of voltage-gated Ca^2+^ channels. Calcium ions (Ca^2+^) enter the presynaptic neuron via these channels, triggering the release of neurotransmitters from synaptic vesicles. These neurotransmitters are discharged through pores into the synaptic cleft at the interface between presynaptic and postsynaptic neurons. Neurotransmitters attach to neuroreceptors on the dendrites of postsynaptic neurons and subsequently enter to initiate signaling in the postsynaptic neuron [77]. The binding of neurotransmitters affects the postsynaptic neuron by either eliciting excitement or inhibition of the action potential.

## 3. Classification and Functionalities of Low-Dimensional Nanohybrids

Low-dimensional nanohybrids (LDNHs) have emerged as powerful platforms for neurochemical sensing and modulation, leveraging the unique properties of 0D, 1D, and 2D nanomaterials to achieve highly sensitive, selective, and biocompatible interfaces with the brain [2]. These nanohybrids, which combine materials across different dimensionalities, enable multifunctional properties that make them particularly well-suited for applications in neurotechnology, where high precision, flexibility, and adaptability are crucial [78]. The integration of different nanomaterials in hybrid systems enhances their overall performance by combining their strengths, resulting in materials that are not only more effective but also more versatile.

**Figure 3:**
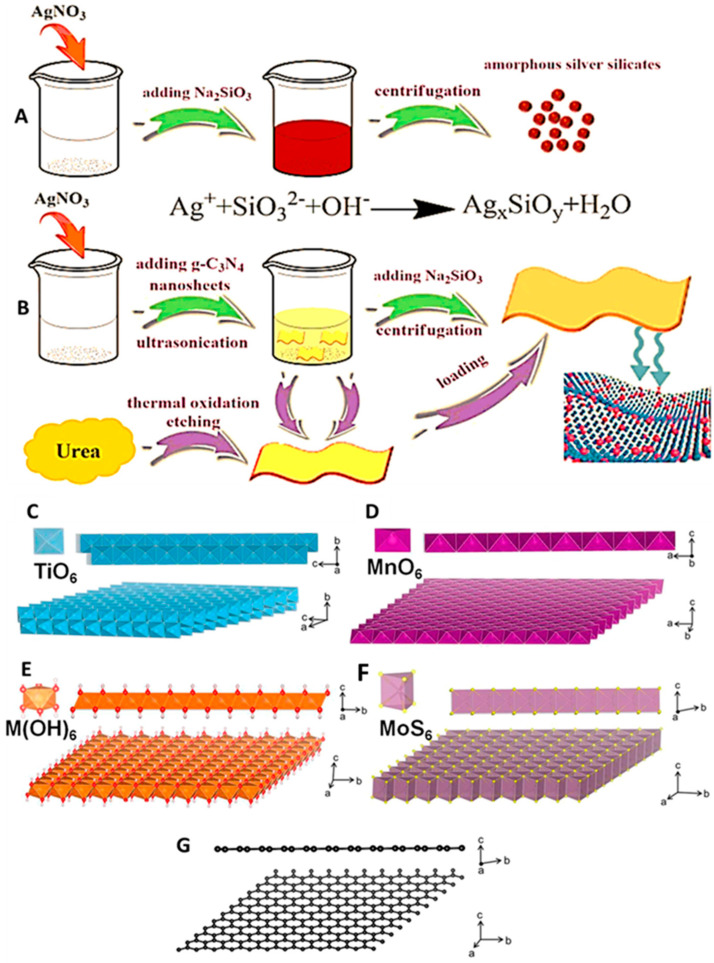
Schematic representation of the synthesis of (**A**) α-AgSiO and (**B**) α-AgSiO/CNNS composites [89]. Schematic structural representations of (**C**) lepidocrocite-type layered titanate, (**D**) MnO_6_ (**E**) layered double hydroxide (LDH), (**F**) molybdenum hexasulfide (MoS_6_), and (**G**) graphene nanosheets. Reproduced with permission from reference [90]. Copyright © 2014, American Chemical Society.

Zero-dimensional (0D) nanomaterials, such as quantum dots (QDs), carbon dots (CDs), and metallic nanoparticles (NPs), are typically characterized by their confined size, which leads to unique optical properties, including size-dependent fluorescence and high quantum yield [79]. Quantum dots, in particular, have been widely utilized for neurochemical sensing due to their excellent photostability, high brightness, and tunable emission spectra. These properties make them ideal for real-time in vivo detection of neurotransmitters and other neurochemicals, enabling high-resolution imaging of dynamic brain processes [80]. Carbon dots and graphene quantum dots (GQDs) offer additional advantages, such as low toxicity, aqueous solubility, and biocompatibility, which are critical for their application in biological systems. Metallic nanoparticles, including gold and silver, are often incorporated into electrochemical and optical biosensing due to their plasmonic properties, which allow for enhanced sensitivity in detection methods such as surface-enhanced Raman spectroscopy (SERS) and electrochemical sensors [81]. However, 0D nanomaterials alone can sometimes face challenges like aggregation in biological environments and limited conductivity, which can hinder their performance in certain applications. One-dimensional (1D) nanomaterials, such as carbon nanotubes (CNTs), nanowires, and nanorods, offer superior electrical conductivity and mechanical strength, making them ideal candidates for applications that require efficient charge transfer and structural integrity [82]. CNTs, both single-walled and multi-walled, are particularly valued in neural engineering because they exhibit exceptional electrical conductivity and are mechanically robust and flexible. These properties are advantageous in neural interfaces, where they enable a high charge injection capacity and low impedance for stable neural signal detection and stimulation [83]. Moreover, CNTs can be functionalized to achieve high specificity and sensitivity for neurotransmitter detection, making them valuable tools for electrochemical biosensors for real-time neurochemical monitoring [83]. Similarly, nanowires, such as those made from silicon or metal oxides, can be used to fabricate field-effect transistors (FETs) or resistive sensors, enabling the label-free detection of neurotransmitters and other biomarkers with high sensitivity. However, the stability of these 1D nanomaterials in physiological environments, as well as their solubility and biocompatibility, must be addressed to ensure their effectiveness in long-term neural applications [84]. Two-dimensional (2D) nanomaterials, such as graphene, molybdenum disulfide (MoS_2_), black phosphorus (BP), MXenes, and graphitic carbon nitride (g-C_3_N_4_), have garnered significant attention for their remarkable electronic, optical, and mechanical properties, which are highly desirable for neurochemical sensing and neuromodulation. These materials exhibit large surface areas and high carrier mobilities, making them ideal for applications that require high sensitivity, fast response times, and minimal interference with biological systems. Graphene, in particular, is a widely studied material due to its excellent conductivity, mechanical strength, and ease of functionalization, which enable efficient interaction with neurotransmitters and other biological molecules [85]. Graphene oxide and reduced graphene oxide, with their tunable surface chemistry, further enhance the potential for enzyme immobilization, molecular recognition, and fluorescence quenching, making them suitable for developing advanced biosensors. Other 2D materials, such as MoS_2_ and MXenes, are gaining traction due to their semiconducting properties, which enable their use in photodetectors and electrochemical sensors for neurotransmitter detection [86]. MXenes, in particular, have shown great promise for use in electrochemical biosensors because of their metallic conductivity, redox activity, and hydrophilic surface terminations, which facilitate their integration into flexible and wearable sensors. Additionally, BP and g-C_3_N_4_ are emerging as promising materials owing to their biodegradability, visible-light activity, and chemical stability, which make them suitable for photoelectrochemical applications in neurochemical sensing [87]. One of the key advantages of LDNHs is their ability to hybridize materials with different dimensionalities, creating synergistic effects that significantly enhance their performance [88]. Reduced particle sizes, improved dispersion, and increased heterojunction interfaces can enhance the photocatalytic efficacy of photocatalysts. Ultradispersed amorphous silver silicate/ultrathin g-C_3_N_4_ nanosheet heterojunction composites (a-AgSiO/CNNS) exhibiting intimate interfacial coupling were synthesized via the straightforward in situ precipitation of ultrafine a-AgSiO (~5.2 nm) uniformly distributed across the entire surface of hierarchical ultrathin CNNS. Ultrathin CNNS serve both as a substrate for heterostructure formation and as a dispersion to inhibit the aggregation of a-AgSiO nanoparticles [89]. 2D nanosheets of layered inorganic solids, synthesized through soft-chemical exfoliation, serve as efficient building blocks for hybridization with inorganic, organic, biological, and polymeric molecules or nanostructures (Figure 3A,B) [90]. Compared to graphene nanosheets, 2D inorganic nanosheets exhibit significantly greater tunability in their chemical composition and physical properties, resulting in the emergence of unforeseen new functions through hybridization. Despite the distinctive and captivating benefits of inorganic nanosheets, research on inorganic nanosheet-based hybrid materials remains limited (Figure 3C–G). The exfoliation of layered transition metal oxides can only be achieved through the intercalation of bulky amine molecules or the ion exchange of ammonium cations into the protonated derivatives of the original materials; thus, the interlayer alkali metal ions of the host materials must first be exchanged with protons or hydronium ions. The exchange of alkali metal ions for protons is accomplished by the straightforward dispersion of unaltered materials in aqueous solutions of strong acids. The resultant protonated materials are treated with bulky organic cations to substitute the interlayer protons or hydronium ions with these larger cations.

For example, the combination of 0D quantum dots with 2D graphene or graphene oxide can result in highly sensitive fluorescence and electrochemical sensors that benefit from both the fluorescence properties of the QDs and the excellent conductivity of the graphene matrix. This hybrid system reduces QD aggregation, improves signal transduction, and enhances sensor stability, allowing for a more reliable and long-lasting performance in biological applications. Similarly, the integration of 1D CNTs with biocompatible polymers, such as polypyrrole or PEDOT:PSS, results in composites that not only possess the high electrical conductivity of CNTs but also offer flexibility and mechanical compliance, making them suitable for implantation in soft tissues. These CNT-polymer hybrids can be used to create highly sensitive neural electrodes capable of monitoring neurotransmitter levels in real-time while maintaining the biocompatibility required for long-term use. Another promising hybridization strategy is the combination of MXenes with hydrogels, which yields flexible, conductive, and biocompatible materials that can conform to the soft tissue of the brain while maintaining stable electrochemical performance for neural interfaces. The hydrophilic nature of MXenes enhances their dispersibility in biological media and promotes better interaction with neural tissues, while the hydrogel matrix provides mechanical support and facilitates the delivery of neurochemicals to specific brain regions. The functional properties of LDNHs, including their electronic, optical, electrochemical, surface chemistry, flexibility, and biocompatibility, are key to their success in neurochemical sensing and modulation. The unique electronic properties of LDNHs, such as their high carrier mobility and low impedance, enable fast and efficient charge transfer, making them ideal for real-time electrochemical measurements and neural stimulation. Their optical properties, including tunable fluorescence and plasmonic effects, are exploited in advanced optical biosensing techniques such as fluorescence microscopy and surface-enhanced Raman spectroscopy (SERS). The electrochemical activity of LDNHs, particularly in materials like MXenes and metal nanoparticles, allows for the amplification of neurochemical signals, improving the sensitivity and detection limits of sensors. Surface functionalization plays a critical role in enhancing selectivity toward specific analytes, such as neurotransmitters, by enabling the attachment of recognition elements like antibodies, enzymes, or aptamers. The flexibility and mechanical compliance of LDNHs also ensure their seamless integration into wearable or implantable systems, making them suitable for the continuous, long-term monitoring of brain activity. Furthermore, the biocompatibility of these materials ensures that they can interface with neural tissues without inducing significant immune responses or toxicity, which is essential for developing devices that can be safely used by humans. The versatility of LDNHs, achieved through strategic hybridization and tuning of material properties, positions them as transformative tools in the fields of neurochemical sensing and neuromodulation. By combining the best characteristics of various nanomaterials, LDNHs enable the development of advanced systems for real-time, non-invasive monitoring and precise modulation of neurochemical activity, paving the way for next-generation neurotechnologies and personalized treatments for neurological disorders.

## 4. LDNHs in Neurochemical Sensing

### 4.1. Electrochemical Sensing

Electrochemical sensing has revolutionized neurochemical monitoring by enabling real-time, sensitive, and selective detection of neurotransmitters. Low-dimensional nanohybrids (LDNHs), which combine the unique properties of nanomaterials like graphene, carbon nanotubes (CNTs), MXenes, quantum dots (QDs), and conducting polymers, have significantly enhanced the performance of these sensors. Their high surface-to-volume ratio, exceptional electrical conductivity, and catalytic activity make them ideal for detecting neurochemicals at ultra-low concentrations. This section explores the role of different LDNHs in electrochemical sensing, focusing on their mechanisms, advantages, and applications in neuroscience.

**Figure 4:**
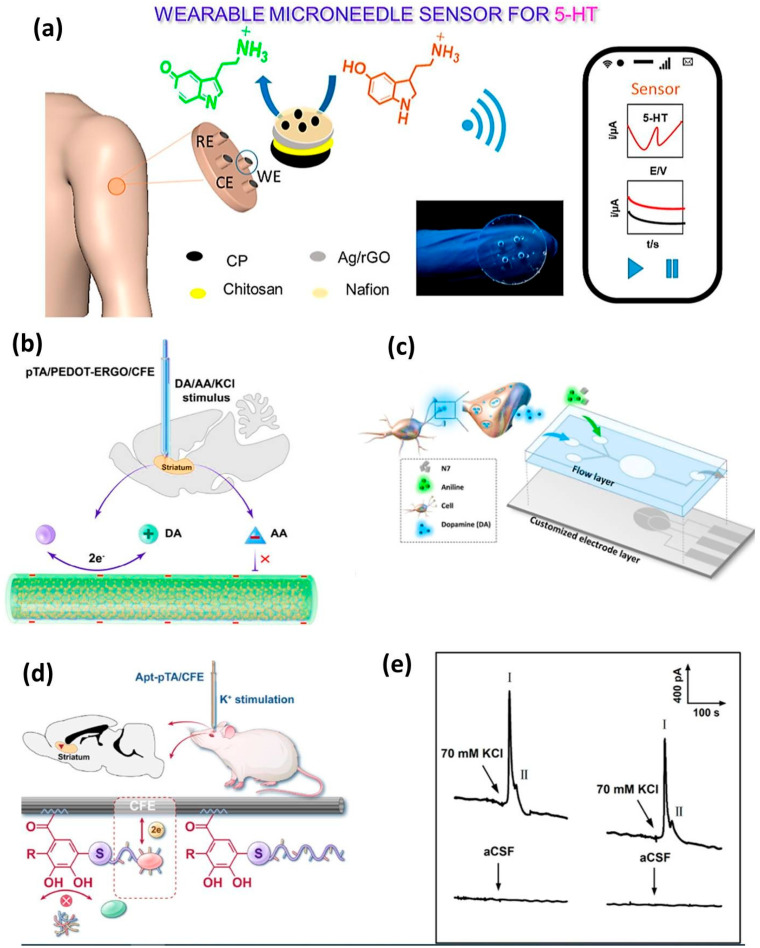
(**a**) Wearable graphene-silver-chitosan nanocomposite electrochemical microneedle sensors for real-time continuous serotonin monitoring. Reproduced with permission from reference [93]. Copyright © 2023, American Chemical Society. (**b**) Schematic illustration of selective DA sensing by pTA/PEDOT-ERGO/CFE in the rat striatum stimulated by local injection of DA, AA, and high K^+^ solution. Reproduced with permission from reference [94]. Copyright © 2024, American Chemical Society. (**c**) Illustration of the layout of an integrated microfluidic biosensor for monitoring DA release from neurons. This integrated chip is composed of a flow and a custom electrode layer. Reproduced with permission from reference [95]. Copyright © 2023, American Chemical Society. (**d**) Schematic illustration of Apt-pTA/CFE for in vivo DA sensing. (**e**) Real-time sensing of Apt-pTA/CFE in the striatum during local injection of 70 mM KCl. Potential: 0.1 V (vs Ag/AgCl). Upon KCl stimulation, the current initially rises sharply, generating spike I within 30 s, followed by a rapid return to baseline as dopamine is metabolized. The stimulus-induced dopamine concentration was determined to be 6.5–7.6 μM, based on the calibration equation [I (nA) = 0.16 CDA (μM) + 0.06, r = 0.9998]. Interestingly, a reproducible shoulder peak (spike II, 1.0–1.2 μM) appears during the current decay, resembling the kinetic profile of neurotransmitter release from vesicles through exocytosis. Reproduced with permission from reference [96]. Copyright © 2024, American Chemical Society.

Graphene and its derivatives, such as reduced graphene oxide (rGO), are widely used in electrochemical sensors due to their high electron mobility, large surface area, and excellent electrocatalytic properties. Functionalized graphene hybrids, such as graphene-metal nanoparticle (Au and Pt) composites, enhance neurotransmitter detection by facilitating electron transfer and reducing oxidation overpotentials. For instance, graphene-Au nanorod hybrids have demonstrated exceptional sensitivity for dopamine (DA) detection, with a limit of detection (LOD) of 0.2 nM in biological fluids [91]. Additionally, nitrogen-doped graphene (N-Gr) improves the selectivity by minimizing the interference from ascorbic acid (AA) and uric acid (UA), which are common challenges in neurochemical sensing. Recent advances include 3D porous graphene foams, which provide a larger active surface area for neurotransmitter adsorption, enabling multiplexed detection of DA, serotonin (5-HT), and norepinephrine (NE) in a single assay. Furthermore, graphene-based field-effect transistors (FETs) have been employed for label-free, real-time monitoring of glutamate, a key excitatory neurotransmitter, with femtomolar sensitivity [92]. Wearable microneedle-based electrochemical sensor technologies are rapidly advancing due to their distinctive features, including low invasiveness, painlessness, and real-time continuous monitoring. Severe disruptions or imbalances in neurotransmitter systems are linked to numerous chronic diseases and mental disorders, including Parkinson’s disease, depression, anxiety, memory impairment, substantial weight fluctuations, and persistent physical or emotional stress. The compelling benefits of wearable microneedle-based sensors provide continuous monitoring of diverse biomarkers and pharmacological agents in interstitial fluid (ISF) [93]. In a separate study, researchers created a sensor for the continuous monitoring of the biologically significant neurotransmitter serotonin (5-hydroxytryptamine, 5-HT) using a microneedle patch (Figure 4a). The novel microneedle sensor array utilizes Ag/rGO-modified carbon paste microneedle electrodes for square wave voltammetry and amperometric detection of the 5-HT target. This real-time orthogonal sensing provides distinct and unique information with the requisite analytical performance. The working electrode surfaces were studied using electroanalytical and surface morphological approaches. The proposed sensor successfully detected the 5-HT target in phosphate-buffered saline within linear ranges of 3–21 and 6–60 μM, exhibiting clear linearity in both short and long ranges. A biocompatible and implantable microsensor exhibiting superior performance was developed through the sequential electrodeposition of poly(3,4-ethylenedioxythiophene)-electrochemically reduced graphene oxide (PEDOT-ERGO) nanocomposites and poly(tannic acid) (pTA) on the surface of a carbon fiber electrode (CFE), demonstrating its viability for in vivo electrochemical sensing applications (Figure 4b) [94]. The synergistic electrocatalytic effect of PEDOT-ERGO nanocomposites combined with negatively charged pTA on the dopamine (DA) redox reaction enables the microsensor to achieve high detection sensitivities of 1.1 and 0.37 nA μM^−1^ within the detection ranges of 0.02–0.5 and 0.5–20 μM, respectively, with a low limit of detection of 9.2 nM.

**Figure 5:**
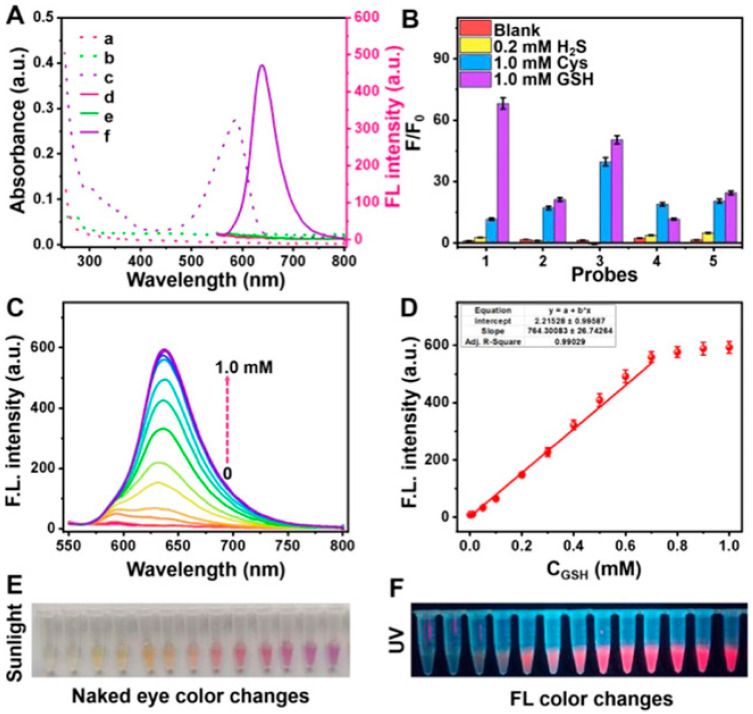
(**A**) UV–visible spectra (a–c) and fluorescence emission spectra (d–f) at λex 488 nm of GSH (a,d), Re-DNP (b,e), Re-DNP + GSH (c,f) solutions. (**B**) Fluorescence responses of five compounds (probes 1–5) to 0.2 mM H_2_S, 1.0 mM Cys, and 1.0 mM GSH for 30 min in 0.1 M PBS (pH 7.4, 5% MeCN, 37 °C). (**C**) Fluorescence response of 10 μM Re-DNP as a function of GSH concentration (0–1.0 mM) in 0.1 M PBS (pH 7.4, 5% MeCN, 37 °C). (**D**) Fluorescence enhancement vs. GSH concentration (10 μM to 0.7 mM). Probe 1 color change (**E**) and fluorescence change (**F**) at varying GSH concentrations (left to right) under visible or UV light at 365 nm. Reproduced with permission from reference [101]. Copyright © 2025, American Chemical Society.

A novel functionalized microneedle electrode is constructed using 4-mercaptophenylboronic acid (4-MPBA) and a polymeric additive (acid chrome blue K, ACBK) to establish a dual-recognition molecular imprinted interface. DA molecules may facilitate an optimal configuration for efficient electron transfer through cyclic borate interactions between the boric acid group of anchoring 4-MPBA and the hydroxyl group, while ACBK should be progressively electropolymerized around the DA molecules, adhering to the electrode surface [97]. The acquired interface featuring an imprinted cavity exhibited a high selectivity for dopamine (DA) molecules, likely due to the dual-recognition site of 4-mercaptophenylboronic acid (4-MPBA) and the complementary shape of the cavity, facilitating an unimpeded electron-transport pathway via 4-MPBA and gold nanoparticles (AuNPs) for the bound DA. The characteristics of the fabricated microneedle electrodes are assessed using electrochemical methods. Thakur et al. highlighted current advancements in nanomaterials utilized for the ultrasensitive detection of dopamine and cholesterol, thoroughly examining their electrochemical activities in relation to heightened sensitivity. Future perspectives and obstacles in detection, together with potential solutions, are examined, and the current market is evaluated. A thorough literature assessment reveals opportunities for enhancing the downsizing of cholesterol and dopamine biosensors in lab-on-chip systems, as well as addressing existing technical constraints to enable complete patient use at home [98]. Integrated microfluidic biosensors that can sensitively detect dopamine in mass-limited samples are therefore of considerable significance. This study involved the development and thorough characterization of a microfluidic biosensor functionalized with a hybrid material consisting of indium phosphate and polyaniline nanointerfaces for dopamine detection (Figure 4c). This biosensor has a linear dynamic sensing range from 10^−18^ to 10^−11^ M and a limit of detection (LOD) of 1.83 × 10^−19^ M during flowing operation [95]. This microfluidic sensor demonstrated not only high sensitivity but also excellent selectivity for DA and remarkable stability, exceeding 1000 cycles. Chen et al. devised a universal covalent grafting technique for attaching an aptamer to a carbon fiber microelectrode (CFE) to selectively quantify dopamine in vivo. The universal strategy involves the oxidation of poly(tannic acid) (pTA) to generate an oxidized form (pTAox), followed by the coupling of a nucleophilic sulfhydryl molecule from the dopamine-binding mercapto-aptamer with the o-quinone moiety of pTAox, utilizing click chemistry for the interfacial functionalization of the CFE surface [96]. The suggested universal method effectively grafted the aptamer onto a glassy carbon electrode, as confirmed by the use of electroactive 6-(ferrocenyl) hexanethiol as a redox reporter (Figure 4d,e). An amperometric approach utilizing the constructed aptasensor for the quantification of dopamine was devised. The aptasensor exhibited a linear range for dopamine detection of 0.2–20 μM, with a sensitivity of 0.09 nA/μM and a detection limit of 88 nM (S/N = 3). We propose a pretreatment approach, effervescent solid-phase extraction (ESPE), which facilitates the effective enrichment of trace analytes for electrochemical detection. In the ESPE process, effervescent tablets composed of gold nanoparticle-decorated graphene oxide (Au/GO) were initially self-dispersed in a test solution to enhance the enrichment of analytes on the Au/GO adsorbents. Subsequently, flocculant effervescent tablets were introduced to induce the formation of self-assembled aggregates of Au/GO sheets, which could then be effectively collected using foam electrodes [99]. The complete sample preparation procedure functioned independently of external power and requires about 5 min. The enhancement mechanism of our approach relies on augmenting the contact probability with analytes via the dynamic dispersion of Au/GO adsorbents, as opposed to the static diffusion process dependent on Brownian motion. We demonstrate that integrating the ESPE solution kit with a portable micro-electrochemical workstation can achieve detection levels equivalent to HPLC in authentic urine samples. Levodopa, the principal treatment drug, requires exact dose because of its limited therapeutic window and intricate pharmacokinetics. This paper introduces the creation of an innovative CuCoFe-LDHzyme-based sweat sensor for the real-time assessment of levodopa levels in patients with Parkinson’s disease [100]. The sensor exhibits exceptional sensitivity and selectivity using differential pulse voltammetry (DPV), with a detection limit of 28.1 nM. The sensor architecture facilitates non-invasive, continuous monitoring, markedly improving patient convenience relative to conventional blood sample techniques. pH correction enables accurate quantification of levodopa in sweat, establishing a robust connection (Pearson coefficient = 0.833) with blood levodopa levels. Glutathione (GSH), an essential antioxidant, is markedly diminished in patients with Parkinson’s disease. This research introduces a dual-mode detection approach for the selective identification of GSH using a single probe. A collection of “turn-on” electrochemical and fluorescent probes was created, utilizing resorufin (Re) as the reporting unit and incorporating specific GSH recognition sites. The 7-(3,5-dinitrophenoxy)-3H-phenoxazin-3-one (Re-DNP) probe was selected for its exceptional selectivity as a fluorescence and electrochemical probe. Its reaction with GSH was markedly superior to that observed with hydrogen sulfide (H_2_S) and cysteine (Cys). The detection limit for glutathione (GSH) using a screen printed carbon electrode (SPCE) modified with carbon nanotubes (CNT) was 5 μM, exhibiting a linear range of 25–500 μM [101]. In fluorescence detection, the probe exhibited a 78-fold enhancement in emission at 630 nm in the presence of GSH, demonstrating a robust linear connection between fluorescence intensity and GSH content within the range of 10–700 μM, with a detection limit of 2 μM (Figure 5). The probe exhibited markedly reduced GSH levels in both PD mice and human patients when tested using actual clinical serum samples, in contrast to healthy controls.

**Figure 6:**
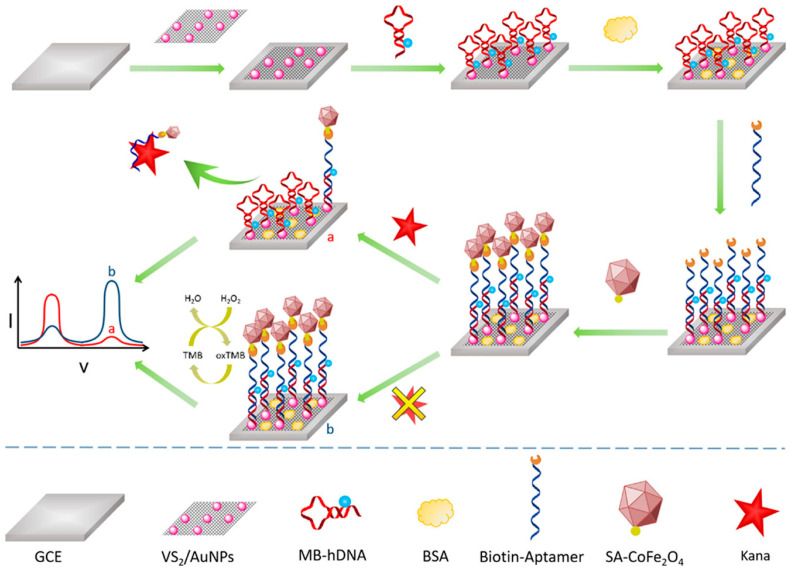
Schematic illustration of the preparation process of the aptasensor and the electrochemical detection strategy of the method. The electrochemical response is illustrated with current (I) plotted against potential (V), where curve a denotes the control signal and curve b represents the enhanced response obtained after target recognition, highlighting the effective detection of Kana. Reproduced with permission from reference [107]. Copyright © 2020, American Chemical Society.

CNTs, particularly single-walled CNTs (SWCNTs), are prized for their high conductivity, mechanical strength, and ability to form nanoscale structures. When hybridized with redox-active materials (e.g., Prussian blue and metalloporphyrins), CNT-based sensors exhibit enhanced electrocatalytic activity toward neurotransmitters. For example, CNT-poly(3,4-ethylenedioxythiophene) (PEDOT) hybrids have been used for ultrasensitive serotonin detection, achieving an LOD of 0.5 nM in human serum [102]. A notable development is the use of vertically aligned CNT (VA-CNT) arrays as nanoscale electrodes, which provide an improved spatial resolution for in vivo brain sensing. These arrays, when functionalized with oxidase enzymes (e.g., glutamate oxidase), enable the selective detection of glutamate in epileptic models [103]. Additionally, CNT-MXene hybrids leverage the metallic conductivity of MXenes and the high surface area of CNTs, achieving picomolar dopamine detection in Parkinson’s disease studies [104]. MXenes (e.g., Ti_3_C_2_T_x_) are a class of 2D transition metal carbides/nitrides with superior metallic conductivity, hydrophilic surfaces, and tunable terminal groups (–O and –F). These properties make them ideal for neurotransmitter sensing, particularly in wearable and implantable devices. For instance, MXene-platinum nanoparticle (PtNP) hybrids have been used for the simultaneous detection of DA and H_2_O_2_ in brain microdialysates, with an LOD of 50 pM [105]. MXenes also excel in FET-based biosensing. A recent study demonstrated that Mo_2_CT_x_ MXene-graphene heterostructures can detect glutamate at 10 fM concentrations, outperforming conventional metal electrodes [106]. Furthermore, the antifouling properties of MXenes make them suitable for long-term in vivo applications, addressing a key limitation of traditional electrochemical sensors. Utilizing the signal amplification properties of planar VS_2_/AuNPs nanocomposites and CoFe_2_O_4_ nanozymes, we developed an electrochemical biosensor for the sensitive measurement of kanamycin (Kana). A ratiometric sensing platform was developed using VS_2_/AuNPs nanocomposites as a support material, characterized by superior conductivity and a high specific surface area, alongside hairpin DNA (hDNA) that facilitates complementary hybridization with biotinylated Kana-aptamer [107]. Furthermore, streptavidin-functionalized CoFe_2_O_4_ nanozymes with enhanced peroxidase-like catalytic activity were mounted onto the aptasensor, enabling the peroxidase-like catalytic reaction to provide amplified electrochemical signals. The presence of Kana led to a quantitative reduction in nanozyme accumulation and enhancement of the methylene blue reaction due to aptamer-biorecognition (Figure 6). Under ideal conditions, the electrochemical signal ratio of the aptasensor exhibited a linear correlation with the logarithmic concentration of Kana, ranging from 1 pM to 1 μM, with a detection limit of 0.5 pM.

Table 1 highlights groundbreaking developments in low-dimensional nanohybrid (LDNH)-enabled electrochemical sensors for neurochemical detection, showcasing their exceptional sensitivity, selectivity, and applicability in neurological research. The MXene-CNT hybrid system achieves an ultra-low detection limit (LOD) of 50 pM for dopamine, which is critical for monitoring Parkinson’s disease, by leveraging the metallic conductivity of MXene and the high surface area of CNTs [108]. For glutamate, the MoS_2_-graphene FET platform demonstrated unparalleled femtomolar (10 fM) sensitivity, enabling real-time tracking of excitatory neurotransmission in epilepsy studies [109]. Graphene-Au nanorod hybrids excel in serotonin detection (0.2 nM LOD), addressing challenges like fouling and interference in complex biofluids [110]. Molecularly imprinted polymers (MIPs) combined with polypyrrole-graphene oxide (PPy-GO) enable norepinephrine sensing at 5 nM in sweat, offering non-invasive stress monitoring [111]. Prussian blue-QD-aptamer sensors achieve a 0.3 nM LOD for GABA, a key inhibitory neurotransmitter, with potential applications in epilepsy diagnostics [112]. These advancements underscore the versatility of LDNHs in tailoring sensor architectures for specific neurochemicals and balancing sensitivity with selectivity [113]. However, challenges such as long-term stability in physiological environments and scalable fabrication remain. Future work should focus on antifouling coatings, machine learning-assisted signal processing, and integration with closed-loop neuromodulation systems to transition these technologies from lab-scale prototypes to clinical and wearable applications. [114] Collectively, these innovations pave the way for precision neurochemical monitoring in both research and healthcare settings.

### 4.2. Optical Sensing (Fluorescence, Raman, SERS, etc.)

**Figure 7:**
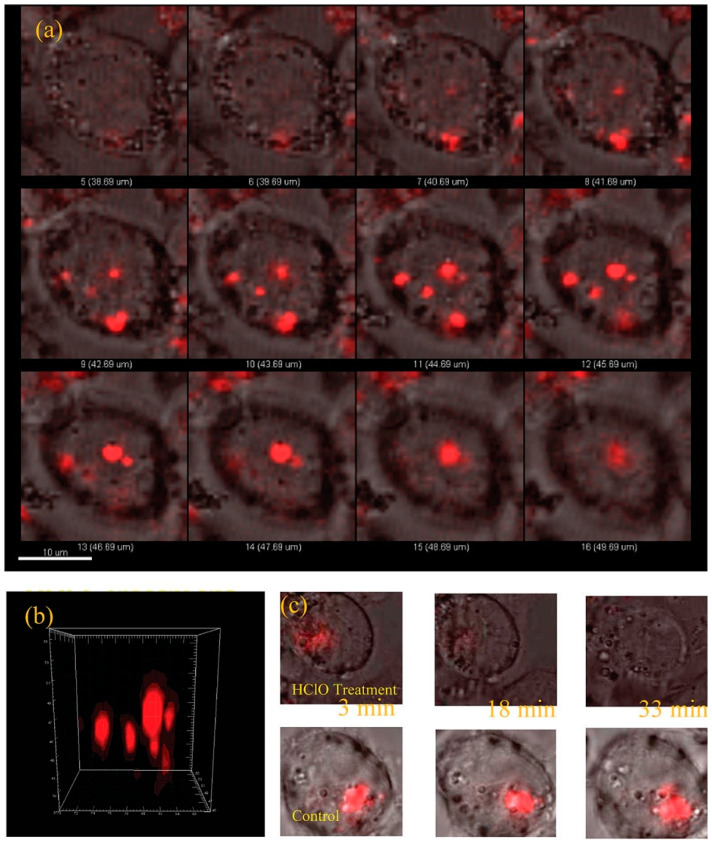
(**a**) Z-stack gallery images of intracellular QDs-poly-CO_2_^−^. The intracellular localization of QDs-poly-CO_2_^−^ was confirmed by scanning optical sections along the z-direction (from bottom to top). The confocal z-stacks were acquired with 1 μm spacing, showing the QDs-poly-CO_2_^−^ distribution at various depths within the cell. (**b**) 3D image of intracellular QDs-poly-CO_2_^−^ in (**a**) was created by assembling the z-stack images from successive focal planes with 1 μm spacing. (**c**) Quenching effect of HClO on the intracellular QDs-poly-CO_2_^−^ (from right to left: 0, 15, and 30 min). Reproduced with permission from reference [119]. Copyright ©2010, American Chemical Society.

Optical sensing techniques, including fluorescence, surface-enhanced Raman spectroscopy (SERS), and Förster resonance energy transfer (FRET), have emerged as powerful tools for neurochemical detection due to their high spatial resolution, multiplexing capability, and compatibility with in vivo imaging. LDNHs, such as near-infrared (NIR) QDs, carbon dot–graphene hybrids, and plasmonic nanoparticles, significantly enhance these optical modalities by improving the signal intensity, photostability, and target specificity. Fluorescent LDNHs, particularly NIR-emitting QDs (e.g., PbS and Ag_2_S), enable deep-tissue neurochemical imaging with minimal autofluorescence interference [118]. For instance, dopamine-sensitive CdSe/ZnS QDs functionalized with boronic acid receptors exhibit fluorescence quenching upon binding, achieving a detection limit of 1 nM in brain extracellular fluid [119]. Four varieties of water-soluble quantum dots (QDs) with distinct organic coating layers were synthesized, and their sensitivities to hypochlorite/hypochlorous acid (HClO) were evaluated. Quantum dots (QDs) with HClO-reactive coatings (methylamino and sulfide groups) demonstrated a protective effect against the HClO-induced quenching of QD fluorescence. In contrast, QDs with hydrocarbon and carboxylate coatings exhibited minimal protection against fluorescence quenching by HClO, resulting in heightened sensitivity for HClO detection. QDs with carboxylate coating layers (QDs-poly-CO_2_^−^) were effectively utilized to determine HClO in tap water. The remarkable selectivity of QDs-poly-CO_2_^−^ for hypochlorite over other reactive oxygen species enabled us to assess myeloperoxidase activity. QDs-poly-CO_2_^−^ was used for hypochlorite detection in HL60 cells via fluorescence imaging (Figure 7).

Dual-mode sensors combining fluorescence and electrochemical detection further enhance reliability; for example, graphene oxide-carbon dot hybrids provide ratiometric fluorescence signals for serotonin while simultaneously enabling voltammetric quantification [2]. FRET-based LDNHs leverage energy transfer between donor-acceptor pairs (e.g., QD-organic dyes) for highly specific neurochemical detection. A CdTe QD–aptamer–Au nanoparticle FRET system was developed for glutamate sensing, where neurotransmitter binding disrupts energy transfer, yielding a 10 nM LOD with millisecond temporal resolution [3]. These platforms are particularly valuable for studying synaptic transmission dynamics.

SERS-active LDNHs, such as Au nanorod-graphene hybrids, amplify Raman signals by several orders of magnitude, enabling label-free identification of neurotransmitters. A recent study demonstrated picomolar (pM) dopamine detection using MXene-Au nanostar SERS substrates with distinct spectral fingerprints in complex biofluids [120]. The resultant superhydrophobic MXene/AuNCs-FOTS membrane not only offers environmental stability to the SERS substrate but also facilitates analyte enrichment, thereby improving sensitivity (LOD = 1 × 10^−14^ M) and reliability (RSD = 6.41%) for Rhodamine 6G (R6G) molecules due to the mitigation of the coffee ring effect. Furthermore, the triadic enhancement mechanism, which integrates plasmonic coupling enhancement from the electromagnetic interactions of adjacent AuNCs in both lateral and longitudinal orientations within the MXene/AuNCs-FOTS membrane, charge transfer from Ti_3_C_2_T_x_ MXene and target molecules, and analyte enrichment capabilities, endows the substrate with exceptional SERS performance and facilitates the precise quantification of biomarkers in urine. Tip-enhanced Raman spectroscopy (TERS) further resolves nanoscale neurotransmitter distribution in neuronal networks [121].

LDNHs like ultrasmall Ag_2_S QDs (λem = 1200 nm) permit non-invasive, real-time glutamate imaging in rodent brains via cranial windows [122]. Challenges remain in improving biocompatibility and minimizing photobleaching; however, advances in polymer-coated SERS probes and two-photon-excited NIR fluorophores are addressing these limitations [123,124,125]. Future directions include wearable optical sensors for point-of-care neurochemical monitoring and closed-loop optogenetic feedback systems that integrate sensing and modulation [126]. A fast synthesis approach for producing monodisperse, biocompatible, lysine-crosslinked mercaptoundecanoic acid (MUA) CdSe_0.25_Te_0.75_/CdS NIR quantum dots is provided, along with their application as probes to investigate long-term in vivo distribution, clearance, and toxicity. These quantum dots exhibit significant signal improvements, facilitating their application as efficient and sensitive probes for live animal imaging. A significant discovery is that mice administered ≈10.5 mg kg^−1^ of NIR QDs via intravenous injection survived for over three months without any observable detrimental effects on their health [127]. The application of nanoparticles in imaging has significantly increased in recent years. This article examines the increasing role of QDs in in vivo imaging of small-animal models. Fluorescent quantum dots, which are small nanocrystals composed of inorganic semiconductor materials, have distinctive optical features that are ideal for in vivo imaging. Quantum confinement effects enable the precise tuning of quantum dot emission color by size, ranging from ultraviolet to near-infrared. Quantum dots exhibit exceptional brightness and photostability [128]. Acute organ injuries, including acute kidney injury (AKI) and acute liver injury (ALI), are associated with high morbidity and mortality rates. Nonetheless, existing clinical treatments are constrained, particularly due to the absence of effective pharmacological interventions. Given that these acute injuries frequently correlate with the overproduction of ROS, developing therapeutic molecules with robust ROS-scavenging capabilities and superior biocompatibility is a promising option for effective antioxidation therapy. Black phosphorus quantum dots (BPQDs), low-dimensional nanomaterials synthesized using a simple one-step process that exhibits fully regulated biodegradation, present considerable potential [129]. This study thoroughly investigated the significant free radical scavenging properties of BPQDs, highlighting their considerable potential in addressing ROS-related organ damage. The interaction between human hemoglobin and economically viable, chemically synthesized CdS QDs (average size ≈ 3 nm) was examined. The semiconductor quantum dots exhibited peak visible absorption at 445 nm, with excitonic production and a band gap of around 2.88 eV, alongside a hexagonal crystalline structure. The binding of QDs-Hb occurs via corona creation, leading to the establishment of a ground-state complex. The durations of heme pocket binding and rearrangement were determined to be t_1_ = 43 min and t_2_ = 642 min, respectively [130]. An effective and straightforward technique for synthesizing nitrogen and sulfur-doped photoluminescent carbon dots (C-dots) was devised to target cancer cells. The development of innovative imaging tools for cancer cells is a crucial and reliable approach to cancer treatment. The fluorescent C-dots synthesized from κ-carrageenan and folic acid can function as effective carriers for marking cancer cells that express folate receptors on their surface. The synthesized C-dots demonstrated significant water solubility, exceptional photostability, and biocompatibility [131].

### 4.3. Quantitative Benchmarks for Sensor Stability and Fouling Resistance

A significant obstacle in the translation of low-dimensional nanohybrid sensors from regulated laboratory environments to practical neurology applications is maintaining long-term stability against biofouling and signal drift [132]. The intrinsic barrier properties of these materials have been qualitatively addressed; however, a quantitative comparison is crucial for assessing their translational potential. The decline in performance within intricate biological matrices, such as serum or cerebrospinal fluid (CSF), is significantly more pronounced than that in idealized buffer solutions. While a sensor may demonstrate merely a 2–5% signal reduction over an hour in phosphate-buffered saline, the same interface can experience a 20–40% decrease in sensitivity when subjected to undiluted serum due to swift protein adsorption [133]. Consequently, evaluating stability using metrics like the percentage of starting current or sensitivity preserved across critical intervals, specifically 1, 12, 24, and 96 h, offers an essential standard for comparing material categories [134]. Moreover, the operating specifications vary considerably according to the application: short-term wearable monitors may accept a sensitivity reduction of less than 10% over 12 h, while chronic implants necessitate significantly higher durability, preferably preserving over 80% of initial performance for several weeks. To address fouling, a quantitative assessment of regeneration protocols, such as electrochemical cleaning cycles capable of restoring 90–95% of the original signal, is essential; nevertheless, the cumulative impact of these operations on the integrity of the nanomaterial must be documented [135]. We recommend a consistent reporting structure that clearly specifies the percentage sensitivity retained after a designated number of cycles (N) or duration (T) in a particular biofluid, coupled with information on any regeneration techniques employed [136]. Setting these quantifiable, application-specific standards will allow for a more stringent assessment of current technologies and inform the systematic design of next-generation nanohybrids that can function reliably in the challenging conditions of the nervous system.

## 5. Multiplexed and Wireless Sensing Systems for Neurochemical Monitoring

The field of neurochemical sensing has undergone a paradigm shift with the advent of multiplexed and wireless sensing systems, which enable real-time, high-fidelity monitoring of neurotransmitter dynamics in both clinical and research settings. These advanced platforms integrate LDNH-based sensors with flexible electronics, wireless communication modules, and implantable devices, overcoming the limitations of traditional wired systems [137]. The convergence of neural dust, wireless micro-electrocorticography (µECoG) arrays, and multimodal implants has opened new frontiers in the understanding of neurological disorders, brain-machine interfaces (BMIs), and personalized medicine [138,139,140]. This section explores the principles, recent breakthroughs, and future directions of these transformative technologies. Epitaxial QDs have achieved on-demand photon generation with great purity, indistinguishability, and brightness; however, they necessitate precision manufacturing and encounter scalability issues. In contrast, colloidal quantum dots are manufactured in batches in solution but generally exhibit larger linewidths, diminished single-photon purities, and unstable emission characteristics. This study presents spectrally stable, pure, and narrow-linewidth single-photon emissions from InP/ZnSe/ZnS colloidal quantum dots [141]. Employing photon correlation Fourier spectroscopy, we detect single-dot linewidths as small as around 5 µeV at 4 K, resulting in a minimum optical coherence time, T2, of around 250 ps. These dots demonstrate negligible spectral diffusion at timeframes ranging from microseconds to minutes and sustain narrow linewidths for durations up to 50 ms, far exceeding those of previous colloidal systems. Furthermore, these InP/ZnSe/ZnS dots exhibit single-photon purities g(2) (τ  =  0) ranging from 0.077 to 0.086 without spectrum filtering. The neural dust platform represents a remarkable miniaturization of neurochemical sensing technology, utilizing submillimeter sensor nodes equipped with LDNH-based detectors. These tiny devices operate through ultrasonic backscatter communication, in which external transducers emit ultrasound pulses that power the implants and receive modulated signals containing neurochemical data. Recent breakthroughs include 300 µm neural dust motes incorporating Prussian blue quantum dot hybrids that successfully monitored dopamine levels in freely moving rodents [142]. While current implementations show great promise, ongoing research aims to further reduce sensor sizes to micrometer scales, which could enable unprecedented single-neuron resolution monitoring of the central nervous system.

## 6. LDNHs in Neuromodulation

### 6.1. Electrical Neuromodulation

Electrical neuromodulation is a powerful technique that involves delivering targeted electrical stimuli to specific brain regions to influence neural activity and restore functional balance in neurological disorders. It has shown remarkable clinical success in treating Parkinson’s disease, epilepsy, chronic pain, and major depressive disorder through modalities such as deep brain stimulation (DBS), vagus nerve stimulation (VNS), and spinal cord stimulation (SCS). However, conventional neuromodulation electrodes, which are typically based on rigid metals such as platinum, iridium, or stainless steel, face significant limitations. Their mechanical mismatch with soft neural tissue can induce inflammation, glial scarring, and eventual loss of function. Furthermore, their large electrode sizes limit spatial resolution, while high impedance reduces stimulation efficiency and selectivity. In this context, LDNHs offer a transformative opportunity to design next-generation neuromodulatory interfaces with nanoscale precision, superior electrical performance, and conformable mechanical properties. LDNHs composed of carbon nanotubes (CNTs), graphene, MXenes, and other conductive nanomaterials can be engineered into ultrathin, flexible, and bioactive electrode platforms. Among them, 1D CNT fibers and films have gained considerable attention due to their extraordinary electrical conductivity, tensile strength, and surface area. When fabricated into microelectrodes or neural threads, CNTs enable a high current injection capacity and significantly lower impedance than traditional materials, allowing more efficient stimulation at lower voltages. These properties minimize electrochemical degradation, reduce tissue damage, and support long-term implantation. Moreover, CNT-based electrodes exhibit high charge storage capacity and excellent signal-to-noise ratios, making them equally suitable for recording and stimulating neuronal activity. Notably, their nanostructured surfaces facilitate intimate contact with neuronal membranes, promoting effective depolarization while minimizing inflammatory responses. 2D nanomaterials further expand the design space for neuromodulation by offering atomically thin yet mechanically robust platforms with tunable electronic properties. Graphene and MXene-based scaffolds have been used to fabricate ultra-conformable stimulation devices that adhere seamlessly to the brain surface or cortical layers. For example, laser-patterned graphene microelectrodes and Ti_3_C_2_Tx MXene films have demonstrated low impedance and high electrochemical stability in in vivo brain stimulation studies. These 2D nanohybrids are often embedded within elastomeric or hydrogel matrices, creating stretchable and minimally invasive electrode arrays that accommodate brain micromotion and reduce immune activation. Such systems are particularly advantageous for neuromodulation in dynamic environments, such as during locomotion or respiration, where traditional rigid devices often fail.

However, the real innovation lies in the potential of LDNHs to enable closed-loop neuromodulation, a system in which real-time sensing of neurochemical or electrophysiological signals dynamically informs the stimulation parameters. This responsive paradigm requires integrated sensors and stimulators with high spatial and temporal resolutions, properties inherently provided by LDNHs. By co-fabricating stimulation electrodes with neurotransmitter sensors on the same flexible platform, using CNTs for stimulation and graphene–enzyme hybrids for sensing, it is possible to construct fully autonomous neural interfaces. These systems can detect aberrant activity or neurochemical imbalance and deliver precise corrective electrical stimulation when and where needed. Such closed-loop configurations have already been explored for adaptive DBS in Parkinson’s disease, seizure prediction in epilepsy, and personalized modulation in treatment-resistant depression. Furthermore, LDNHs support miniaturization and multiplexing, which are essential for interfacing with densely packed and functionally diverse neural circuits. Multi-electrode arrays based on CNTs, graphene, or hybrid composites can stimulate and record data from multiple brain regions simultaneously, enabling a more comprehensive understanding of neural network dynamics and enhancing therapeutic specificity. The scalability of LDNH-based systems also holds promise for the development of high-density wireless neurostimulators that can be implanted with minimal invasiveness, thereby reducing surgical complexity and patient recovery time.

Conventional techniques involve the generation of electric fields adjacent to neural tissues using electrodes or non-invasive modulation employing light, chemicals, magnetic fields, and ultrasound. The emergence of nanotechnology signifies a novel progression in neuro-modulation methods, providing enhanced accuracy and the capacity to selectively target specific cell types. Intelligent nanomaterials facilitate the transformation of distant signals (such as light, magnetic fields, or ultrasound) into localized stimuli (e.g., electric fields or thermal energy) for neurons [143]. Metal ions contribute to Aβ aggregate deposition and neurotoxicity through several mechanisms, including the acceleration of Aβ aggregation, disruption of normal metal homeostasis, and generation of ROS. Despite the potential of metal chelation as a treatment approach for Alzheimer’s disease (AD), its broad application faces a substantial challenge: distinguishing between harmful metals linked to Aβ plaques and those essential for normal metal homeostasis. Moreover, the multiple characteristics of Alzheimer’s disease and the absence of a universally accepted theory to explain its neurodegeneration limit therapy options to a single therapeutic approach. This research introduces an innovative bifunctional platform that combines nonpharmacological and pharmacological stimuli into a single system for the treatment of Alzheimer’s disease. This electrically responsive drug release device, which utilizes a conducting polymer polypyrrole (PPy) integrated with graphene-mesoporous silica nanohybrid (GSN) nanoreservoirs, enables on-demand controlled drug delivery with spatial and temporal precision [144]. Functional materials capable of wirelessly absorbing energy from a specific physical field and subsequently emitting a localized physical signal offer significant benefits in facilitating non-invasive or minimally invasive, precise indirect physical stimulations to enhance therapeutic outcomes for neurological disorders [145].

**Figure 8:**
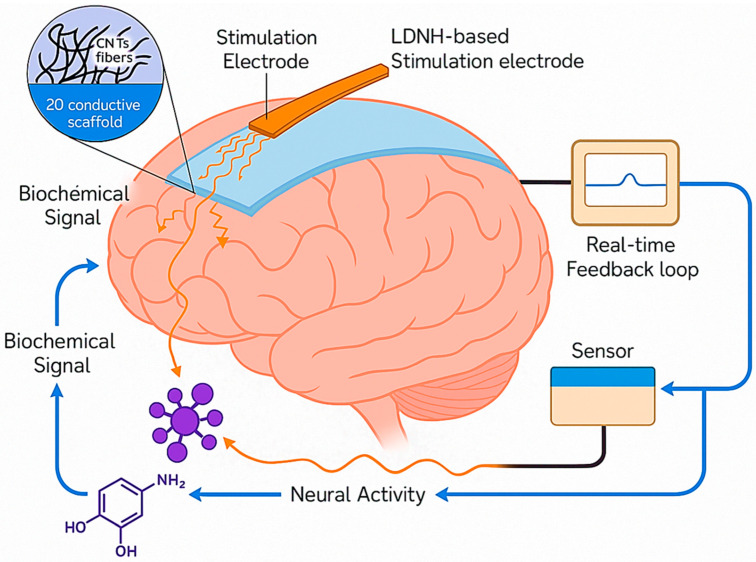
Schematic illustration of closed-loop electrical neuromodulation enabled by LDNHs.

The schematic in Figure 8 illustrates the mechanism of closed-loop electrical neuromodulation using LDNHs, highlighting how these advanced materials enable real-time feedback-responsive brain interfacing. At the core of the system is a flexible LDNH-based stimulation electrode composed of conductive nanomaterials, such as CNT fibers or 2D materials like MXenes or graphene, which conform to the brain’s surface. These nanoscale electrodes deliver precise electrical pulses to targeted neuronal populations, modulating abnormal neural activity in disorders such as epilepsy, Parkinson’s disease, and depression. The stimulation triggers neuronal responses, which alter biochemical signaling pathways represented by neurotransmitter molecules like dopamine or glutamate. These biochemical changes are detected by integrated sensors, which may include enzyme-functionalized graphene or CNT-based electrochemical interfaces that convert neurochemical concentrations into electrical signals. This signal is fed into a real-time feedback loop, where the data are continuously analyzed to adjust the stimulation parameters—intensity, frequency, or location—based on the brain’s dynamic biochemical state. The loop enables closed-loop neuromodulation, ensuring that stimulation is only delivered when aberrant activity or neurochemical imbalance is detected, optimizing therapeutic precision and minimizing side effects. The hybrid material system combines flexibility, high conductivity, and biointerface compatibility, allowing for stable, long-term implantation with minimal immune response. This integrative approach represents a paradigm shift toward intelligent, self-regulating neurotechnologies that harmonize stimulation with the biochemical rhythms of the brain.

### 6.2. Optogenetics and Photo-Modulation

**Figure 9:**
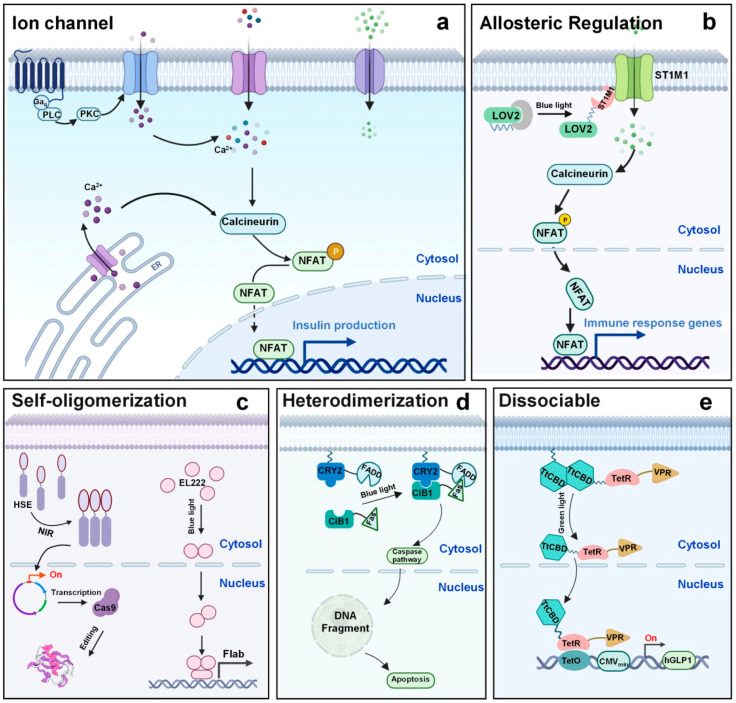
Classification of photoregulatory mechanisms of optogenetic systems. (**a**) Ion channel. (**b**) Allosteric regulation. (**c**) Protein self-oligomerization. (**d**) Protein-target heterodimerization. (**e**) Protein–protein dissociable systems. Reproduced with permission from reference [148]. Copyright © 2024, American Chemical Society.

Optogenetics and photo-modulation technologies have fundamentally reshaped the landscape of neuroscience by offering unparalleled control over neuronal activity with high spatiotemporal precision. These techniques utilize light—typically in the visible or near-infrared (NIR) spectrum—to either directly modulate genetically sensitized neurons or to trigger physicochemical responses in photoreactive nanomaterials. The introduction of low-dimensional nanohybrids (LDNHs) into this domain has dramatically expanded the versatility and functional capabilities of photoresponsive neuromodulation systems. Two upconversion hybrid nanoparticles, designated PT-UCNP-B/G, modified with photothermal agents, were demonstrated to influence neuronal activity through photostimulation and thermo-stimulation under near-infrared laser irradiation at 980 nm and 808 nm, respectively. PT-UCNP-B/G generates visible light (410–500 nm or 500–570 nm) via the upconversion process at 980 nm, while demonstrating an efficient photothermal effect at 808 nm without visible emission or tissue injury [146]. Huang et al. presented a neuro-inspired optical sensor utilizing two-dimensional NbS_2_/MoS_2_ hybrid films, which exhibit exceptional photo-induced conductance flexibility and low electrical energy usage. An optical sensor array inspired by neural mechanisms, comprising 10 × 10 NbS_2_/MoS_2_ phototransistors, facilitates advanced functionalities of sensing, memory, and contrast enhancement for static images, thereby enhancing the image recognition accuracy of convolutional neural networks (CNNs) [147]. Significantly, in-sensor trajectory registration of dynamic light spots was experimentally performed, enabling post-processing to achieve excellent restoration accuracy. Optogenetics, an effective biotechnology that uses light stimulation to bidirectionally regulate protein function, has been extensively utilized in the biomedical sector. Light of varying wavelengths has been employed to modulate the activity of proteins of interest (POI) in mammalian cells, such as activating or silencing neuronal populations, elucidating neural circuits, comprehending the intricate cellular connections of the central nervous system, and addressing neurological disorders. This section primarily summarizes the fundamental action principles and regulatory mechanisms of sample photosensory modules following their incorporation into host protein scaffolds [148]. Ion channels or pumps present in the targeted cell type facilitate membrane depolarization or hyperpolarization upon activation by a specific light (Figure 9). The most commonly utilized opsins are channelrhodopsins (ChRs) and melanopsin, which are responsive to blue light and function as cation and calcium ion channels, respectively. Conversely, halorhodopsins, bacteriorhodopsins (BRs), and archaerhodopsins expel protons or import chloride ions, resulting in membrane hyperpolarization, and have been extensively used to investigate the inhibition of excitable cells.

Unlike traditional optogenetic approaches that rely on the genetic expression of light-sensitive ion channels (e.g., channelrhodopsins), photo-modulation using LDNHs can be implemented in a fully non-genetic, minimally invasive manner. This strategy is particularly valuable for clinical translation because it bypasses the ethical and regulatory challenges associated with gene editing. Instead, LDNHs act as nanotransducers, converting light energy into heat or reactive chemical species that can activate or inhibit nearby neurons via photothermal or photochemical effects. Two-dimensional semiconductors, such as MoS_2_, WS_2_, BP, and MXenes, are especially promising in this context due to their broad and tunable optical absorption spectra, high surface-to-volume ratios, and ability to dissipate absorbed energy efficiently. These properties allow precise control over local thermal gradients or redox environments at the cellular scale without the need for invasive implants or high-intensity light sources. Kaushik et al. identified 34 BLUF photoreceptors derived from bacterial and eukaryotic sources using accessible bioinformatics sequence databases. Our analysis reveals a variety of BLUF-effector configurations with a functional relationship that was previously unrecognized or considered uncommon within the BLUF class of sensory proteins, including endonucleases, the tet repressor family (tetR), regulators of G-protein signaling, the GAL4 transcription family, and several other previously unidentified effectors, such as RhoGEF, Phosphatidyl-Ethanolamine Binding protein (PBP), ankyrin, and leucine-rich repeats. Interaction studies and indexing of BLUF domains further demonstrate the diversity of BLUF-effector combinations [149].

**Figure 10:**
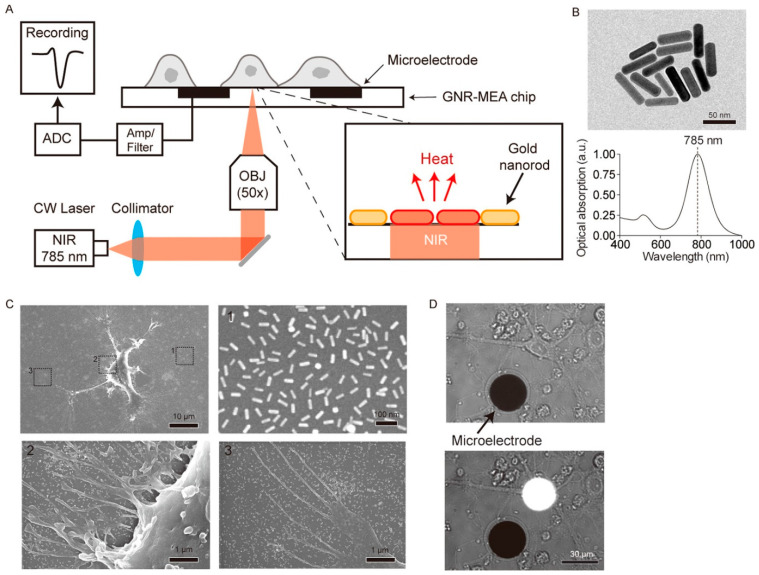
Single-neuron photothermal inhibition. (**A**) Schematic of the photothermal stimulation platform with a GNR monolayer and concentrated NIR light for localized photothermal conversion. (**B**) Top: transmission electron microscopy image of PEGylated GNRs. Bottom: absorbance spectra. (**C**) SEM image of a GNR-integrated glass substrate-cultured neuron. (1) high-magnification image highlighting the PEGylated gold nanorods; (2, 3) enlarged regions showing neuron–nanorod interactions at different contact points. (**D**) Neurons before and after NIR illumination. Reproduced with permission from reference [150]. Copyright © 2018, American Chemical Society.

Photothermal neuromodulation is one of the most well-established mechanisms by which LDNHs influence neuronal function. When irradiated with NIR light, photothermal nanomaterials rapidly convert photon energy into localized heat, leading to transient increases in membrane capacitance or thermal activation of temperature-sensitive ion channels, such as TRPV1. For instance, black phosphorus nanosheets and MoS_2_ nanosheets have been shown to generate localized heating upon 808 nm laser excitation, sufficient to modulate the action potentials of nearby neurons. These effects occur on a sub-second timescale and are spatially confined to micrometer-scale regions, enabling precise targeting of individual cells or subcellular compartments. Notably, 2D nanomaterials can be engineered with biodegradable or responsive coatings to further enhance their biocompatibility, reduce toxicity, and enable repeatable stimulation cycles without inducing inflammation or tissue damage. Yoo et al. devised a nanoplasmonic method to block single-neuron activity with a high temporal resolution. Low-intensity near-infrared light was concentrated at the scale of a single cell on a microelectrode array platform combined with gold nanorods, producing a photothermal effect beneath a designated neuron for photothermal stimulation. The researchers discovered that photothermal stimulation modified the spontaneous activity of a target neuron in an inhibitory manner (Figure 10). Single-neuron inhibition is rapid and exceptionally reliable without causing thermal harm, and it can alter network firing patterns, perhaps indicating its utility for in vivo circuit modulation and functional connectomes [150].

In neural tissues, where thermal sensitivity is tightly coupled to function and viability, this degree of control is essential for safe and effective photo-modulation. In addition to purely thermal effects, certain LDNHs also support photochemical stimulation, in which light exposure triggers catalytic or redox reactions that modulate neuronal signaling. For example, g-C_3_N_4_ nanosheets and metal-doped TMDs can generate reactive oxygen species (ROS) or drive localized ion release in response to visible-light irradiation. These reactions can depolarize neurons by affecting membrane ion gradients or by modifying extracellular microenvironments. Although high levels of ROS can be cytotoxic, carefully controlled doses have been employed therapeutically for the modulation of pain pathways, glioma inhibition, and neurogenesis. Moreover, hybrid systems incorporating plasmonic nanoparticles (e.g., AuNPs) with 2D semiconductors can enhance photochemical activity via local field enhancement, enabling stimulation at lower light intensities or through deeper tissue penetration. These developments open avenues for chemical-free, light-driven neuromodulation tools that operate without electrodes or pharmacological agents, reducing the risk of systemic side effects.

**Figure 11:**
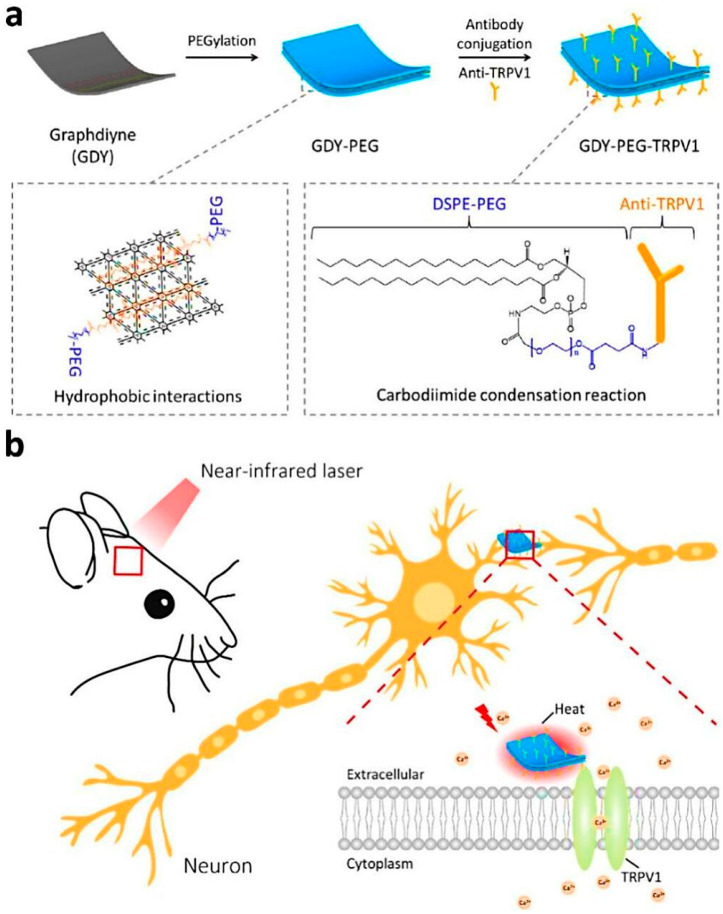
Surface-functionalized GDY as a near-infrared nanotransducer for transcranial photothermal neuromodulation. (**a**) Scheme outlining the preparation of the GDY-based nanotransducer. (**b**) Schematic illustration of the GDY-based nanotransducer for photothermal neuromodulation in living animals under transcranial near-infrared laser illumination. Upon NIR laser exposure, the GDY-PEG-TRPV1 nanotransducer generates localized heat, activating TRPV1 channels on the neuron membrane and inducing calcium (Ca^2+^) influx into the cytoplasm. Reproduced with permission from reference [152]. Copyright © 2024, American Chemical Society.

One particularly exciting frontier enabled by LDNHs is light-triggered site-specific drug delivery, which combines photothermal or photochemical effects with smart nanocarriers to achieve spatially and temporally controlled therapeutic release. In this approach, photoresponsive LDNHs are functionalized with therapeutic payloads, such as dopamine precursors, neurotrophic factors, or anti-inflammatory agents, which are retained under normal conditions and released upon illumination. For example, BP or MXene nanosheets can be incorporated into thermosensitive liposomes or polymeric hydrogels that undergo phase transitions upon NIR heating. By altering ligands on the surface of dendrimers, efficient therapeutic and diagnostic platforms can be developed and utilized for targeted delivery. Dendrimer-based nanocarriers have significant potential for gene delivery. Dendrimers present intriguing packaging and delivery options for central nervous system (CNS) therapies, as enzymes can destroy genetic elements during circulation in the bloodstream. The DNA and RNA enclosed in polyamidoamine dendrimers utilized for targeted brain delivery, through chemical-structural modifications and suitable production, markedly enhance the relationship between transfection efficiency and cytotoxicity. This article provides a thorough review of the structures, synthesis methods, and biological applications of dendrimers [151]. Upon irradiation, the local temperature increase disrupts the carrier matrix, releasing the encapsulated drug into the surrounding neural tissue. This strategy has been demonstrated in preclinical models for localized treatment of neurodegenerative disorders, such as Parkinson’s disease or Alzheimer’s disease, where targeted delivery of levodopa or BDNF is needed to restore function without inducing systemic toxicity. Another approach involves photo-cleavable linkers, where light-sensitive chemical bonds tether drug molecules to the surface of nanohybrids. Upon exposure to a specific wavelength of light, these bonds break, releasing the active compound in a highly localized manner. This strategy enables precision dosing in response to physiological triggers or as part of a closed-loop system, where drug delivery is initiated only when a pathological signal is detected by integrated sensors. These multifunctional systems, often incorporating both photothermal and electrochemical elements, represent a powerful paradigm for responsive neurotherapeutics, offering not only real-time modulation of neural circuits but also on-demand chemical intervention. LDNHs also enable deeper tissue penetration for photo-modulation, addressing one of the major limitations of conventional optogenetics. NIR light (700–1000 nm), particularly in the NIR-II window (1000–1350 nm), can penetrate several millimeters into brain tissue with minimal scattering and absorption. LDNHs that absorb in this region, such as doped TMDs, AuNR composites, and carbon-based hybrids, can serve as effective transducers for deep-brain photostimulation without requiring optical fibers or invasive implantation. This capacity is crucial for modulating the subcortical structures involved in memory, motivation, and arousal, where traditional optical tools are too invasive or imprecise. Recent studies have demonstrated successful non-invasive photoactivation of midbrain dopaminergic neurons and hippocampal circuits using NIR-absorbing LDNHs in combination with systemic or intracranial nanoparticle delivery. Furthermore, the modularity of LDNHs allows for simultaneous integration of diagnostic and therapeutic functionalities, forming the basis of theranostic platforms. For instance, a graphene–gold nanohybrid system can simultaneously image neural inflammation via SERS, sense glutamate concentration electrochemically, and trigger photothermal drug release. This convergence of imaging, sensing, and stimulation capabilities within a single nanohybrid platform can significantly accelerate real-time decision-making in clinical neurointervention, enabling personalized and adaptive treatment regimens. The miniaturization of these multifunctional systems onto flexible or injectable platforms supports long-term implantation with reduced immune response, enabling chronic monitoring and modulation in both research and therapeutic settings. Despite these promising advances, several challenges remain in the clinical translation of LDNH-based photo-modulation systems. Biocompatibility, long-term retention, potential phototoxicity, and immune interactions must be thoroughly addressed through rational surface modifications and dosing protocols. Moreover, the scaling and reproducibility of LDNH synthesis with consistent optical properties remain a technical bottleneck. Regulatory pathways for nanomaterial-based neurotechnologies are still evolving, particularly for hybrid systems that combine devices, drugs, and photonic components. Nonetheless, ongoing research on biodegradable, immuno-inert, and brain-penetrant LDNHs is rapidly advancing the field toward safe and effective clinical applications. Photothermal regulation of neuronal activity is a viable method for elucidating brain circuitry and formulating therapeutics for neurological disorders. The limited neuron selectivity and suboptimal light-to-heat conversion of current photothermal nanomaterials considerably restrict their applicability in neuromodulation (Figure 11a,b) [152]. They demonstrate that graphdiyne (GDY) can be engineered into an effective neuron-targeted photothermal transducer for the in vivo control of neuronal activity via strategic surface functionalization (Figure 11a,b). They functionalized GDY with polyethylene glycol (PEG) via noncovalent hydrophobic interactions and then conjugated antibodies to precisely target the temperature-sensitive transient receptor potential cation channel subfamily V member 1 (TRPV1) on the surface of brain cells. The nanotransducer exhibits excellent photothermal conversion efficiency in the near-infrared spectrum and significant TRPV1-targeting capabilities.

**Figure 12:**
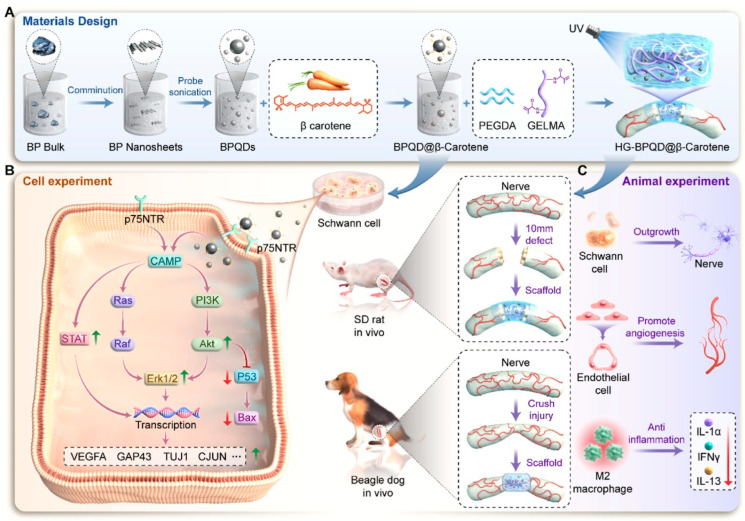
BPQD@ β-carotene-modified BPQDs enhance SC nerve repair in PNI: (**A**) Preparation of BPQD@ β-carotene-embedded GelMA/PEGDA Scaffold; (**B**) Favored neural regrowth, angiogenesis, and inflammatory regulation of SCs; (**C**) Application. Reproduced with permission from reference [153]. Copyright © 2024, American Chemical Society.

Peripheral nerve damage is a significant societal issue. Black phosphorus (BP) has intrinsic advantages over cell-based therapies in regenerative medicine. However, regulating spontaneous degradation and size-dependent cytotoxicity remains challenging, hindering clinical translation. This study involved the fabrication of zero-dimensional BP QDs modified with the antioxidant β-carotene, followed by a thorough investigation of Schwann cells (SCs) to clarify their potential for peripheral nerve regeneration (Figure 12) [153]. In vitro tests revealed that BPQD@β-carotene exhibits negligible toxicity and excellent biocompatibility, promoting brain regeneration, angiogenesis, and the inflammatory control of stem cells. Additionally, the PI3K/Akt and Ras/ERK1/2 signaling pathways were active in SCs at the genetic, protein, and metabolite levels.

## 7. Magnetothermal and Chemical Modulation

**Figure 13:**
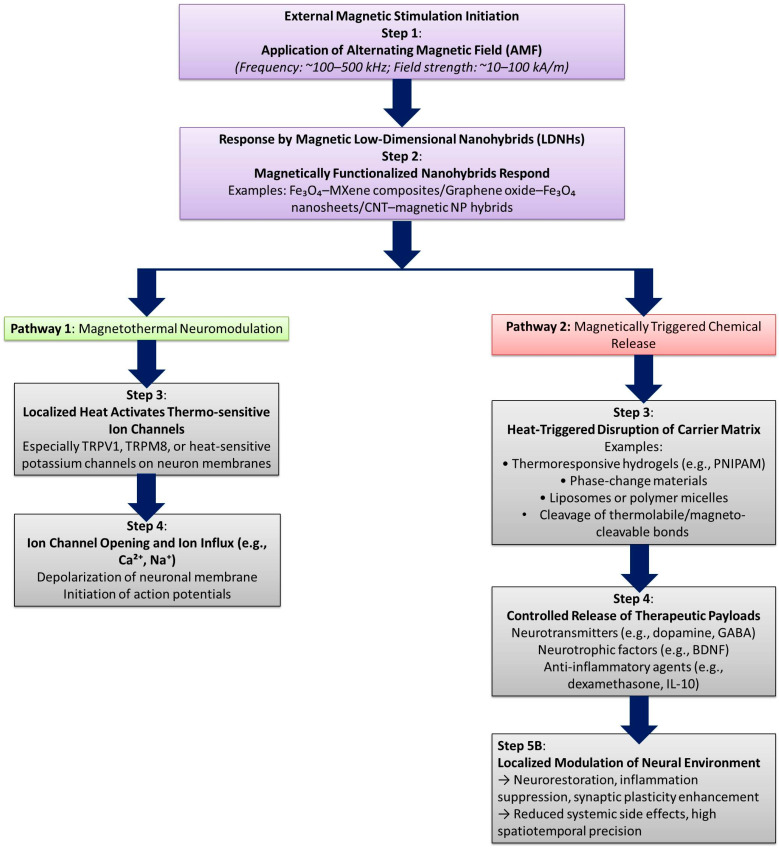
Mechanism of magnetothermal and chemical neuromodulation via magnetically responsive LDNHs.

The principle of magnetothermal neuromodulation relies on the activation of thermosensitive ion channels in neurons, such as TRPV1 or TRPM8, which open in response to localized temperature changes. When magnetic nanohybrids are delivered to specific brain regions and exposed to an external AMF (typically in the 100–500 kHz range), they undergo rapid Brownian and Néel relaxations, generating subcellular-scale heating [154]. This localized temperature increase is sufficient to trigger transient depolarization of neurons through thermal activation of ion channels without damaging surrounding tissue [155]. Notably, LDNHs allow fine-tuning of the heating profile due to their high surface area, controllable magnetic loading, and integration with temperature-sensitive polymeric carriers or 2D scaffolds that enhance thermal confinement and spatial selectivity. Compared to electrical stimulation, this wireless approach avoids mechanical implants and tethered devices, significantly reducing the risks of infection, scarring, and inflammation [156]. In addition to direct neuromodulation, magnetically responsive nanohybrids can facilitate the on-demand, site-specific release of neuromodulators, anti-inflammatory agents, and neurotrophic factors. These systems typically consist of a magnetic core (e.g., Fe_3_O_4_ NPs) encapsulated within a thermoresponsive matrix, such as poly(N-isopropylacrylamide) (PNIPAM), or tethered to drug molecules via thermolabile or magneto-cleavable linkers [157,158,159]. Upon AMF exposure, localized heating disrupts the carrier matrix or cleaves the chemical bond, releasing the therapeutic payload into the extracellular space [160]. Such delivery systems have shown promise in preclinical models for the treatment of Parkinson’s disease, where dopamine or its precursors can be selectively released in the striatum in response to behavioral stimuli. Similarly, anti-inflammatory cytokines (e.g., IL-10 or dexamethasone) have been magnetothermally delivered to regions affected by neuroinflammation, offering controlled pulsatile treatment without systemic exposure. The use of 2D LDNHs further enhances their capabilities. MXenes, graphene derivatives, and layered double hydroxides (LDHs) can be co-engineered with magnetic nanomaterials to form composite structures that combine magnetic responsiveness with a high drug-loading capacity, electrical conductivity, and tissue-compliant mechanical properties. For instance, Fe_3_O_4_–graphene oxide hybrids have been used for magnetothermal heating, while simultaneously offering ROS-scavenging capabilities and facilitating cell adhesion. In addition, functional groups on 2D surfaces enable the precise conjugation of bioactive molecules, improving targeting and minimizing off-target effects. These smart systems not only enable the remote control of chemical delivery but also create opportunities for multimodal interfaces that combine thermal, electrical, and biochemical cues for advanced neural modulation strategies. Based on the mechanism outlined in Figure 13, magnetothermal and chemical neuromodulation using magnetically responsive LDNHs represents a transformative approach for the wireless, minimally invasive control of neural activity. Upon application of an alternating magnetic field (AMF), embedded magnetic nanoparticles—typically iron oxide—within the LDNH matrix absorb energy and generate localized heat through Brownian and Néel relaxation processes [161]. This heat can activate thermosensitive ion channels, such as TRPV1, on neuronal membranes, triggering membrane depolarization and inducing action potentials. The spatial precision and tunable heating capacity of LDNHs enable targeted neural stimulation with reduced tissue damage and without the need for physical implants. Simultaneously, this thermal effect can serve as a stimulus for controlled drug release. LDNHs integrated into thermoresponsive carriers or designed with magneto-cleavable linkers allow heat to disrupt the carrier matrix or cleave chemical bonds, resulting in site-specific release of neuromodulators or anti-inflammatory agents. This dual-mode capability enables both acute neuromodulation and sustained chemical interventions on the same platform. Furthermore, integration with real-time sensing modules supports closed-loop systems that can autonomously adjust stimulation or drug delivery in response to biochemical cues [162,163,164]. Collectively, this mechanism underscores the potential of LDNH-based systems to enable precise, adaptable, and patient-specific neuromodulation therapies with reduced invasiveness and systemic exposure.

## 8. Biointerface Engineering, Brain Integration and Artificial Intelligence (AI)

The seamless integration of low-dimensional nanohybrids (LDNHs) into neural systems represents a transformative frontier in neurotechnology, merging biointerface engineering with AIto revolutionize diagnostics, therapy, and brain-machine interfaces [165]. Central to this integration is the development of advanced materials and strategies that minimize immune rejection, enable targeted delivery across the blood–brain barrier (BBB), and ensure long-term functional stability within the brain’s delicate microenvironment [166]. Simultaneously, the rise of AI-driven neurotechnologies poses profound ethical questions regarding brain privacy, cognitive enhancement, and equitable access [166]. This section explores cutting-edge strategies for optimizing brain-LDNH interfaces, the current status of clinical translation, and the ethical implications of these rapidly advancing technologies.

**Figure 14:**
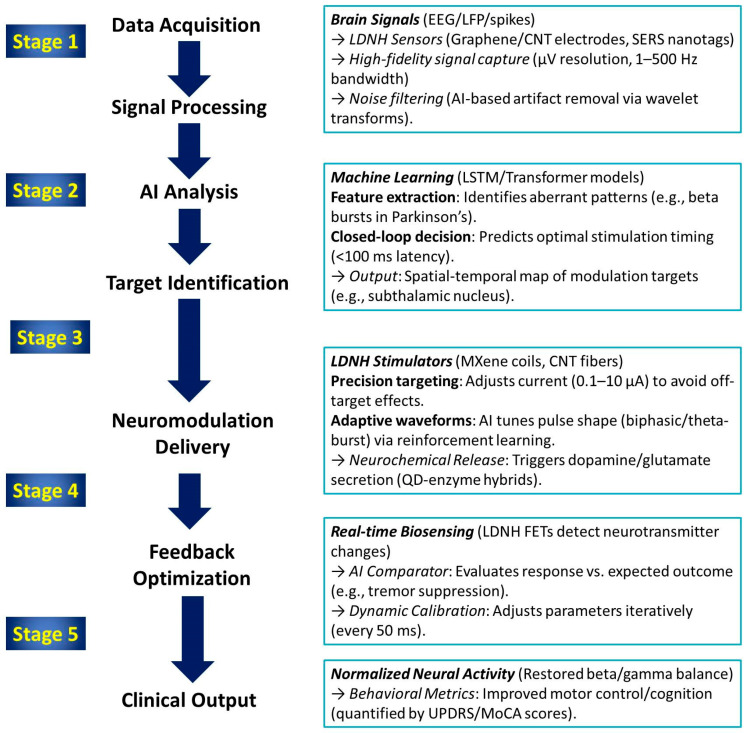
AI-enhanced LDNH brain neuromodulation model.

One of the most significant challenges in deploying LDNHs for neuromodulation and sensing is mitigating the foreign body response, which can lead to chronic inflammation, glial scar formation, and eventual device failure [167]. The brain’s immune system, specifically microglia and astrocytes, swiftly encases implanted materials in dense fibrotic tissue, therefore isolating the device and impairing its functionality. In response, researchers have developed various bioengineering solutions (Figure 14). Soft and flexible LDNH composites that replicate the mechanical properties of neural tissue (Young’s modulus ~1–10 kPa) substantially diminish inflammation caused by mechanical mismatch. Graphene-polyethylene glycol (PEG) hydrogels have tissue-like flexibility and excellent electrical conductivity, facilitating stable neuronal interfacing without inducing glial activation. Moreover, surface functionalization with anti-inflammatory agents, such as interleukin-4 (IL-4) or dexamethasone-loaded nanoparticles, effectively inhibits microglial polarization toward pro-inflammatory phenotypes [168]. Recent advances in zwitterionic polymer coatings, such as poly(2-methacryloyloxyethyl phosphorylcholine) (PMPC), have demonstrated near-complete resistance to protein fouling and glial adhesion in rodent models, preserving electrode functionality for more than a year. Another promising approach involves bioactive coatings that release neurotrophic factors (e.g., brain-derived neurotrophic factor, BDNF) to promote neuronal integration while inhibiting scar-forming astrocytes [169]. These strategies collectively aim to create “immune-stealth” interfaces, where LDNHs operate undetected by the host’s defense mechanisms.

The brain’s dynamic mechanical environment is subject to pulsations, movements, and growth, which demand neural interfaces that can withstand mechanical stress without losing functionality [170]. Conventional stiff electrodes frequently fail due to shear-induced injury or delamination from adjacent tissues. Conversely, LDNHs embedded within viscoelastic polymers (such as polydimethylsiloxane, PDMS, and silk fibroin) provide flexible, self-healing conductors that preserve electrical continuity even at a 30% strain [171]. For instance, carbon nanotube (CNT)-embedded gelatin hydrogels combine high conductivity (~1000 S/cm) with tissue-mimetic compliance, enabling uninterrupted recordings during brain motion. Similarly, MXene-polyacrylamide hybrids have been used in cortical surface arrays that conform to gyral curvatures without inducing trauma [172]. These materials are augmented by mesh-like structures created using sacrificial layer methods, enabling ultraflexible electronics to be administered through minimally invasive needles before growing to encompass extensive regions of the cortex. Such designs are essential for epilepsy monitoring grids and motor prosthetics, where enduring stability is crucial. Recent research emphasizes dynamic LDNH networks that adjust to morphological alterations in developing brains (e.g., pediatric applications) or degenerative tissues (e.g., Alzheimer’s disease models), where traditional implants would rapidly become incompatible with their mechanical surroundings [173].

Beyond invasive implants, LDNHs have been engineered to traverse the BBB for non-invasive diagnosis and therapy. The tight junctions of the BBB traditionally block > 98% of systemically administered drugs and nanomaterials; however, LDNHs functionalized with BBB-shuttling ligands (e.g., transferrin and angiopep-2) exploit receptor-mediated transcytosis for efficient brain uptake. Quantum dots (QDs) coated with apolipoprotein E (ApoE) mimic low-density lipoprotein (LDL) particles, achieving >10-fold higher brain accumulation than unmodified nanoparticles [174,175]. Once across the BBB, LDNHs can be further targeted to specific cell types using peptides (e.g., rabies virus glycoprotein-derived peptides for neurons) or antibodies (e.g., anti-GFAP for astrocytes) [176]. Magnetoelectric LDNHs, such as cobalt ferrite-coupled graphene oxide, enable spatiotemporal control via external magnetic fields, releasing drugs precisely at epileptic foci or tumor sites [177]. These platforms are being tested for neurodegenerative diseases; for example, cerium oxide-loaded mesoporous silica nanoparticles reduce reactive oxygen species in Parkinson’s disease models while simultaneously delivering dopamine agonists [178]. However, long-term biodistribution and nanotoxicity concerns necessitate rigorous preclinical profiling, particularly of metallic components that may accumulate in peripheral organs.

The clinical translation of LDNH-based neurotechnologies has achieved notable milestones, particularly in neuroprosthetics and deep brain stimulation (DBS) [179]. Wireless graphene-based DBS systems are in Phase I/II trials for Parkinson’s disease, demonstrating improved energy efficiency and reduced side effects compared with conventional metal electrodes. Similarly, CNT-enhanced Utah arrays have achieved >5-year stability in human motor cortex trials for tetraplegic patients, enabling typing speeds of 90 characters per minute using brain–computer interfaces. In epilepsy, MXene-coated grids are being evaluated for intraoperative mapping, where their high signal-to-noise ratio improves the localization of seizure foci. Despite these advances, several key hurdles persist [180]. Long-term stability studies have revealed that even flexible LDNHs face gradual performance decay due to oxidative degradation (e.g., graphene) or enzymatic cleavage (e.g., polymer coatings). Regulatory agencies now require exhaustive genotoxicity and neuroinflammation data, particularly for nanomaterials that may fragment over time [181,182,183]. The recent FDA approval of the first partially biodegradable LDNH implant (a silk-Mg neural interface) sets a precedent for transient devices but underscores the need for a standardized degradation protocol.

As LDNH-AI systems grow more sophisticated and capable of decoding thoughts, emotions, and intentions, ethical frameworks struggle to keep pace. The potential for “brain hacking” via wireless implants raises alarms about data privacy; neural signals can be intercepted or manipulated, necessitating quantum-level encryption for neurodata [184]. Cognitive enhancement poses another dilemma: Should LDNH-augmented memory or learning be restricted to medical rehabilitation or allowed for healthy individuals seeking competitive edges? Current debates center on equity, as such technologies may exacerbate societal divides if they are accessible only to affluent populations. International consortia like the NeuroRights Initiative advocate for legal protections against coercive neurotechnology use, informed by lessons from genomics and AI ethics. Meanwhile, the militarization of neurotech, such as DARPA’s efforts to enhance soldier cognition, demands urgent oversight to prevent unethical applications [185,186].

## 9. Strategies for Reducing Immune Response, Glial Scar Formation

**Figure 15:**
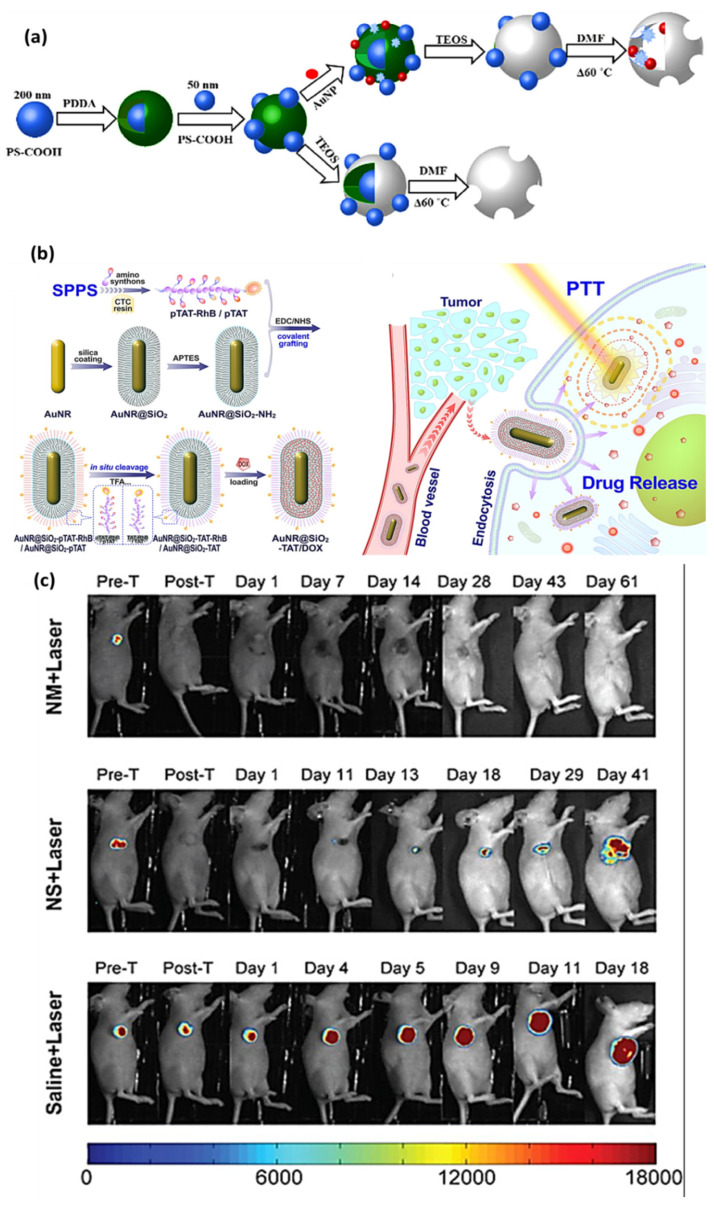
(**a**) The figure shows the formation of hollow silica nanogolf balls (HGBs) and gold-embedded HGBs. PDDA coating over 200 and 50 nm carboxylate-functionalized polystyrene assembled the template nanoparticles. The template was then coated with silica or linked to gold nanoparticles. Subsequently, DMF is used to etch the silica-coated template and remove the polystyrene template. Synthetic Procedures for AuNR@SiO_2_ Nanocomposites. Reproduced with permission from reference [194]. Copyright © 2017, American Chemical Society. (**b**) Application in Synergistic Chemo-Photothermal Cancer Therapy. Reproduced with permission from reference [195]. Copyright © 2020 American Chemical Society. (**c**) Evaluation of tumor response to photothermal therapy using bioluminescence imaging. The bioluminescence signal is generated only in living cancer cells due to luciferase activity. Representative mice from each experimental group showing luciferase activity in the tumor. The mice injected with nanomatryoshkas or nanoshells and treated with laser experienced loss of bioluminescence in the area illuminated by the laser, as observed after therapy. Mice were euthanized when the tumor volume reached 1500 mm^3^ or if the tumor persisted for 60 days after treatment. Reproduced with permission from reference [197]. Copyright © 2014, American Chemical Society.

The integration of LDNHs into neural systems requires overcoming three critical challenges: immune rejection, mechanical mismatch, and blood–brain barrier (BBB) penetration [187]. Recent advances in biointerface engineering have yielded innovative solutions to these obstacles, leveraging materials science and nanotechnology to enable stable, high-performance neural interfaces [188]. The foreign body response to implanted LDNHs, characterized by microglial activation, astrocytic scarring, and fibrotic encapsulation, remains a major barrier to chronic stability. Research has demonstrated that surface chemistry and topography are pivotal in modulating immune reactions. For instance, zwitterionic polymer coatings (e.g., poly(sulfobetaine methacrylate)) reduce protein fouling by >90%, significantly delaying the encapsulation of microglia. Similarly, nanostructured surfaces that mimic the extracellular matrix (e.g., graphene oxide nanowrinkles) promote neuronal adhesion while suppressing astrocyte proliferation. Pharmacological strategies, such as the localized release of anti-inflammatory agents (dexamethasone-loaded mesoporous silica nanoparticles), have shown efficacy in rodent models, extending electrode functionality from weeks to over a year [189]. A breakthrough study reported that conducting hydrogels with dynamic covalent bonds (e.g., boronate ester-crosslinked PEDOT) resist fibrous scarring by adapting to tissue remodeling and maintaining impedance stability for 18 months post-implantation. Conventional rigid electrodes mechanically mismatch brain tissue (Young’s modulus: ~1 kPa), causing chronic inflammation and signal degradation. To address this issue, ultrasoft LDNH composites have been engineered to match neural mechanics. For example, CNT-embedded gelatin hydrogels exhibit tissue-like elasticity (E ≈ 2 kPa) while retaining high conductivity (~1000 S/m). These materials maintain their functionality under a 30% strain, which is critical for accommodating brain pulsations. Recent work introduced self-healing MXene-silk fibroin hybrids that autonomously repair mechanical damage, ensuring uninterrupted operation in dynamic environments [190]. Mesh electronics, fabricated via sacrificial layer techniques, enable minimally invasive delivery through needles before expanding to conform to cortical contours, as demonstrated in a human-compatible μECoG array. Such designs have reduced glial scarring by 70% compared to their rigid counterparts in primate trials [191]. Non-invasive LDNH delivery across the BBB is essential for treating neurodegenerative diseases. Strategies exploiting receptor-mediated transcytosis (e.g., transferrin-conjugated quantum dots) achieve brain uptake efficiencies of ~5% ID/g, which is 10 times higher than that of untargeted nanoparticles. Magnetoelectric LDNHs (e.g., CoFe_2_O_4_-graphene hybrids) enable spatiotemporal control under external magnetic fields, precisely releasing drugs at amyloid plaques in Alzheimer’s models [192]. For glioblastoma, angiopep-2-functionalized MXenes selectively accumulate in tumors, enhancing the efficacy of photothermal therapy while sparing healthy tissue [193]. However, the long-term nanotoxicity of metallic components requires rigorous evaluation. Another study highlighted that ultrasmall (<5 nm) silica-coated gold clusters exhibit renal clearance, addressing accumulation concerns [194]. To elucidate their cellular absorption and imaging capabilities, Au@SiO_2_ HGBs were infused with QDs (Figure 15a). Confocal fluorescence microscopy and atomic force microscopy images demonstrated the consistent endocytosis of QD-loaded Au@SiO_2_ HGBs in adhering HeLa cells and circulating red blood cells (RBCs). Surface-enhanced Raman spectroscopy of Au@SiO_2_ hollow gold nanobeads in red blood cells demonstrated an increased strength of the Raman signal corresponding to the spectral markers peculiar to the red blood cell membrane. Li et al. utilized mesoporous silica-coated gold nanorods (AuNR@SiO_2_) as a theranostic platform for synergistic chemotherapy and photothermal cancer treatment [195]. Employing an in situ grafting–cleavage approach, the cell-penetrating TAT peptide (YGRKKRRQRRR) was covalently attached to the silica surface of AuNR@SiO_2_ nanocomposites and subsequently activated through conventional cleavage treatment to produce AuNR@SiO_2_-TAT drug nanocarriers directly. FT-IR spectroscopy and pronounced polarity alterations demonstrated the efficacy of various peptide modification techniques (Figure 15b). TAT-modified drug nanocarriers showed substantial improvements in intracellular absorption, acidic endolysosome internalization, and passive tumor accumulation. A photo-theranostic agent utilizing chlorin e6 (Ce_6_) photosensitizer-conjugated silica-coated gold nanoclusters (AuNCs@SiO_2_–Ce_6_) is meticulously developed and synthesized for fluorescence imaging-guided photodynamic treatment (PDT). The AuNCs@SiO_2_–Ce_6_ exhibits the following characteristics: (i) elevated loading of Ce_6_ photosensitizer; (ii) absence of non-specific Ce_6_ release during circulation; (iii) markedly improved cellular uptake efficiency of Ce_6_, resulting in substantially enhanced photodynamic therapeutic efficacy relative to free Ce_6_; (iv) subcellular characterization of the nanoformulation through both Ce_6_ fluorescence and plasmonic luminescence of AuNCs; and (v) fluorescence imaging-guided photodynamic therapy (PDT) [196]. Ayala-Orozco et al. conducted a direct comparison investigation of approximately 90 nm diameter gold nanomatryoshkas (Au/SiO_2_/Au) and approximately 150 nm diameter gold nanoshells about their photothermal therapeutic efficacy in extremely aggressive triple-negative breast cancer (TNBC) tumors in mice. Au nanomatryoshkas have a robust light absorption efficiency of 77%, whereas nanoshells have a comparatively lower absorption efficiency of about 15%. Following intravenous administration of Au nanomatryoshkas and a subsequent single NIR laser application at 2 W/cm^2^ for 5 min, 83% of the TNBC tumor-bearing mice exhibited health and were tumor-free over 60 days, whereas only 33% of mice treated with nanoshells survived for the same duration (Figure 15c). The reduced dimensions and enhanced absorption cross-section of Au nanomatryoshkas render this nanoparticle more efficacious than Au nanoshells for photothermal cancer therapy.

## 10. Outlook and Future Perspectives

The future of localized dynamic nanohybrids (LDNHs) in neuromodulation lies in the intriguing convergence of modern materials science, artificial intelligence (AI), and bioelectronics. The integration of LDNHs into brain–computer interfaces (BCIs) and closed-loop neurotechnologies signifies a transformative direction, as neuroscience increasingly requires precision, adaptability, and customization. These technologies provide not only passive monitoring but also active, real-time regulation of brain circuits, ushering in a new era of individualized neuro-nano-medicine.

The simultaneous use of LDNHs with AI-powered closed-loop systems presents considerable potential for dynamic and autonomous neuromodulation. Nanohybrid systems can be programmed to provide on-demand therapeutic interventions, such as light, heat, magnetic fields, or targeted medication release, by integrating cognitive algorithms that analyze electrophysiological and biochemical signals in real time. For example, magnetothermal or optothermal LDNHs can be activated upon the identification of disease signals, such as epileptic discharges or neuroinflammatory markers, facilitating precisely timed interventions. AI not only improves responsiveness but also facilitates long-term adaptive learning, thereby increasing system intelligence over time, reducing side effects, and optimizing therapeutic benefits.

**Figure 16:**
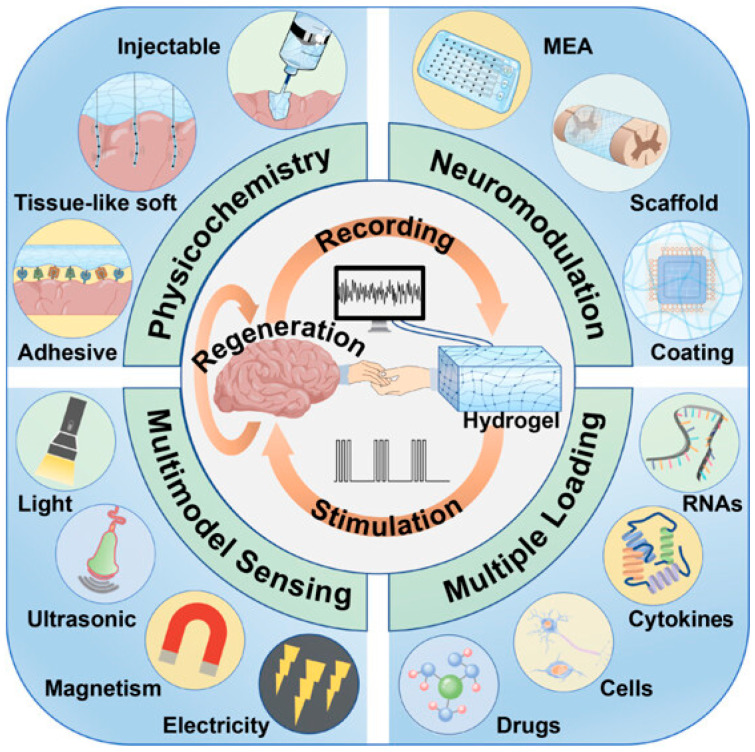
Functional components of controllable neuromodulation for translational therapeutics. Reproduced with permission from reference [198]. Copyright ©2025 American Chemical Society.

In brain–computer interfaces, low-dimensional nanohybrid structures present a viable avenue for minimally invasive, multifunctional interactions with neural tissues. In contrast to conventional rigid electrodes, soft nanoengineered materials provide enhanced mechanical compliance, less foreign body response, and multifunctional attributes, including biosensing, imaging, and therapeutic applications. By integrating photoactive, piezoelectric, or magnetoresponsive elements, LDNHs can facilitate the recording of brain activity and modulation of specific regions with exceptional spatial precision. The dual function of recording and stimulating significantly improves the effectiveness of closed-loop BCIs in prosthetics, neurorehabilitation, and neuropsychiatric therapies. The advent of personalized medicine necessitates the use of instruments that are precisely tailored to individual brain architectures and disease patterns. LDNHs, characterized by adjustable surface chemistry, multifunctional payload delivery, and reactivity to external stimuli, offer a diverse platform for precise treatment. Carbon-based nanohybrids infused with therapeutic medicines or diagnostic markers can be designed to specifically address neurotransmitter imbalances or inflammatory pathways. The future will likely involve a combination of patient-specific neuroimaging and omics data to create customized nanostructures for certain brain regions and cell types. Controllable neuromodulation utilizing multimodal triggers combined with hydrogels constitutes a revolutionary treatment approach for pro-regenerative brain repair. The strategic integration of programmable neuromodulatory treatments and hydrogels designed for specific neuronal niches is essential for clinical use, as it is characterized by reduced invasiveness and enhanced therapeutic effectiveness (Figure 16). This review clarifies the physicochemical characteristics of hydrogels, categorizes hydrogel-based neuromodulation into five specific modes (electrical, ionic, biomechanical, optical, and biochemical), and emphasizes the inherent multidimensional structural and chemical engineering utilized to improve neuromodulatory efficacy.

Despite these advancements, significant challenges persist. A significant impediment to their clinical use is the scalability and reproducibility of intricate LDNH systems. Long-term biocompatibility and possible neurotoxicity must be meticulously evaluated, particularly in chronic implantation or recurrent injection. Accessing deep tissue continues to be difficult, especially for optically or magnetically driven systems, as tissue dispersion and attenuation restrict the efficacy of external manipulation in deep brain areas. Overcoming these obstacles requires synchronized initiatives in material innovation, sophisticated imaging, regulatory structures, and interdisciplinary cooperation. A critical consideration for the translational application of low-dimensional nanohybrids in neurochemical sensing and modulation is their biocompatibility and chronic stability, which vary significantly across material classes. Carbon-based nanohybrids, particularly graphene oxide and carbon nanotubes, often exhibit excellent biostability but can provoke foreign body responses depending on their surface charge, functionalization, and aspect ratio. Coating with biocompatible polymers like Nafion or polyethylene glycol (PEG), is a common strategy to mitigate this and to reduce glial scarring. In contrast, conductive polymer-based hybrids, such as PEDOT:PSS, generally demonstrate superior soft tissue compatibility and flexibility, minimizing mechanical mismatch with neural tissue, although concerns regarding the leaching of acidic dopants or residual monomers necessitate rigorous long-term testing. Metal and metal-oxide nanohybrids (e.g., those incorporating Pt, Au, or MnO_2_) offer exceptional catalytic performance but face significant challenges related to ion leaching and potential neurotoxicity, which must be addressed through stable encapsulation or alloying. Ultimately, the chronic stability of any implant is a function of the immune response and the material’s resistance to biofouling and degradation in a corrosive physiological environment. A direct comparison reveals a critical trade-off: while carbon- and metal-based systems often provide superior electrochemical performance, polymer-based hybrids frequently lead to biocompatibility. Therefore, the choice of material must be application-specific, balancing the need for sensitivity and longevity with the imperative to minimize foreign body response and ensure long-term safety. This underscores the necessity for standardized, long-term in vivo studies to directly compare these key parameters across different material platforms.

The integration of LDNHs with AI, BCIs, and closed-loop systems represents a transformative advancement in neuromodulation and neural-interface technologies. Ongoing advancements in these systems may facilitate the development of highly tailored, adaptable, and less invasive treatments for a broad range of neurological disorders.

## 11. Conclusions

Localized dynamic nanohybrids (LDNHs) are a groundbreaking advancement in neurosensing and neuromodulation. By integrating the multifunctionality of nanomaterials with the adjustability of external stimuli—such as light, heat, magnetism, and electric fields—LDNHs offer an unparalleled degree of control over brain interfaces. Their capacity to react to localized physiological signals, administer targeted therapeutic agents, and regulate neural activity with precise temporal and spatial resolution represents a notable advancement over conventional neurotechnology models that depend on broad stimulation or pharmacological treatments. In neurosensing, LDNHs provide real-time and highly sensitive identification of brain biomarkers, neurotransmitters, and localized biochemical conditions. This facilitates dynamic feedback and closed-loop control in neurological diagnosis and therapy. In neuromodulation, non-invasive or minimally invasive activation enables the precise targeting of neuronal circuits while reducing off-target effects, tissue damage, and systemic toxicity. The dual capability of monitoring and intervention establishes LDNHs as a fundamental element in next-generation neurotherapeutics. The integration of LDNHs with innovative technologies like artificial intelligence, brain–computer interfaces, and soft bioelectronics could transform the field of precision neurotechnology. Data-driven closed-loop systems enable nanohybrids to be directed by real-time feedback, facilitating the adaptive and tailored control of brain circuits. This presents promising opportunities for the treatment of intricate neurological disorders, including epilepsy, Parkinson’s disease, depression, and traumatic brain injury, with enhanced efficacy and reduced adverse effects. The future vision for neurotechnologies involves creating fully integrated, multifunctional, and individualized platforms that can interact seamlessly with the central nervous system. These systems integrate the sensitivity and specificity of LDNHs with computational intelligence, biocompatibility, and scalability for clinical implementation. Achieving this ambition necessitates addressing critical hurdles, such as material stability, long-term safety, precise deep-tissue targeting, and the need to obtain regulatory approval.

In conclusion, LDNHs provide a revolutionary framework for deciphering, interacting with, and ultimately addressing brain-related disorders. Their adaptability, precision, and integration capabilities signify a pivotal advancement in the development of individualized neuro-nano-medicine. As diverse research converges, these intelligent nanohybrids are set to connect neurology, engineering, and clinical care—heralding a new era of responsive, intelligent, and personalized neurotechnologies.

## Figures and Tables

**Table 1:** Key advances in LDNH-based neurochemical sensing.

Neurotransmitter	LDNH System	Detection Method	LOD	Key Application	Ref.
Dopamine (DA)	MXene-CNT hybrid	Amperometry	50 pM	Parkinson’s disease monitoring	[108]
Glutamate	MoS_2_-graphene FET	FET-based sensing	10 fM	Epilepsy research	[109]
Serotonin (5-HT)	Graphene-Au nanorods	DPV	0.2 nM	Depression biomarker detection	[25]
Norepinephrine (NE)	PPy-MIP-GO	Impedimetry	5 nM	Stress monitoring in sweat	[111]
GABA	Prussian blue-QD-aptamer	Redox cycling	0.3 nM	Epilepsy diagnostics	[112]
Dopamine	PEDOT:PSS-MoS_2_	CV	0.8 nM	Neurodegenerative disease studies	[113]
Serotonin	CNT-PEDOT	Amperometry	0.5 nM	Psychiatric disorder analysis	[114]
Glutamate	MXene-PtNP	Chronoamperometry	50 pM	Brain injury monitoring	[115]
Dopamine	CdTe QD-tyrosinase	Photoelectrochemistry	0.1 nM	Neuropharmacology research	[116]
Norepinephrine	ZnO-CuO nanofibers	EIS	5 nM	Wearable stress sensors	[117]

## Data Availability

Not applicable.

## Supplementary Materials

The following supporting information can be downloaded at: https://www.mdpi.com/article/10.3390/biom15101405/s1, Figure S1: PRISMA 2020 flow diagram illustrating the process of study identification, screening, eligibility assessment, and inclusion in the systematic review
