# Supplementary material for: A Review on Low-Dimensional Nanoarchitectonics for Neurochemical Sensing and Modulation in Responsive Neurological Outcomes
Source: Biomolecules. 2025 Oct 2;15(10):1405. doi: 10.3390/biom15101405 (PMC12562191; doi:10.3390/biom15101405)
Supplement: Supplementary file 1 [file biomolecules-15-01405-s001.zip › biomolecules-3868084-supplementary.pdf]

Supporting information of

A Review on Low-Dimensional Nanoarchitectonics for Neurochemical Sensing and Modulation in Responsive Neurological Outcomes

Mohammad Tabish [1,2,*], Iram Malik [2,3], Ali Akhtar [4] and Mohd Afzal [5,*]

1 Department of Pharmacology, College of Medicine, Shaqra University, Shaqra 11961, Saudi Arabia
2 King Salman Center for Disability Research, Riyadh 11614, Saudi Arabia
3 Department of Electrical Engineering, College of Engineering, Prince Sattam bin Abdulaziz University,
Al-Kharj 11942, Saudi Arabia; i.malik@psau.edu.sa
4 Department of Pharmacognosy, College of Pharmacy, King Saud University, Riyadh 11451, Saudi Arabia; aakhtar@ksu.edu.sa
5 Department of Chemistry, College of Science, King Saud University, Riyadh 11451, Saudi Arabia
* Correspondence: tabish@su.edu.sa (M.T.); maslam1@ksu.edu.sa (M.A.)

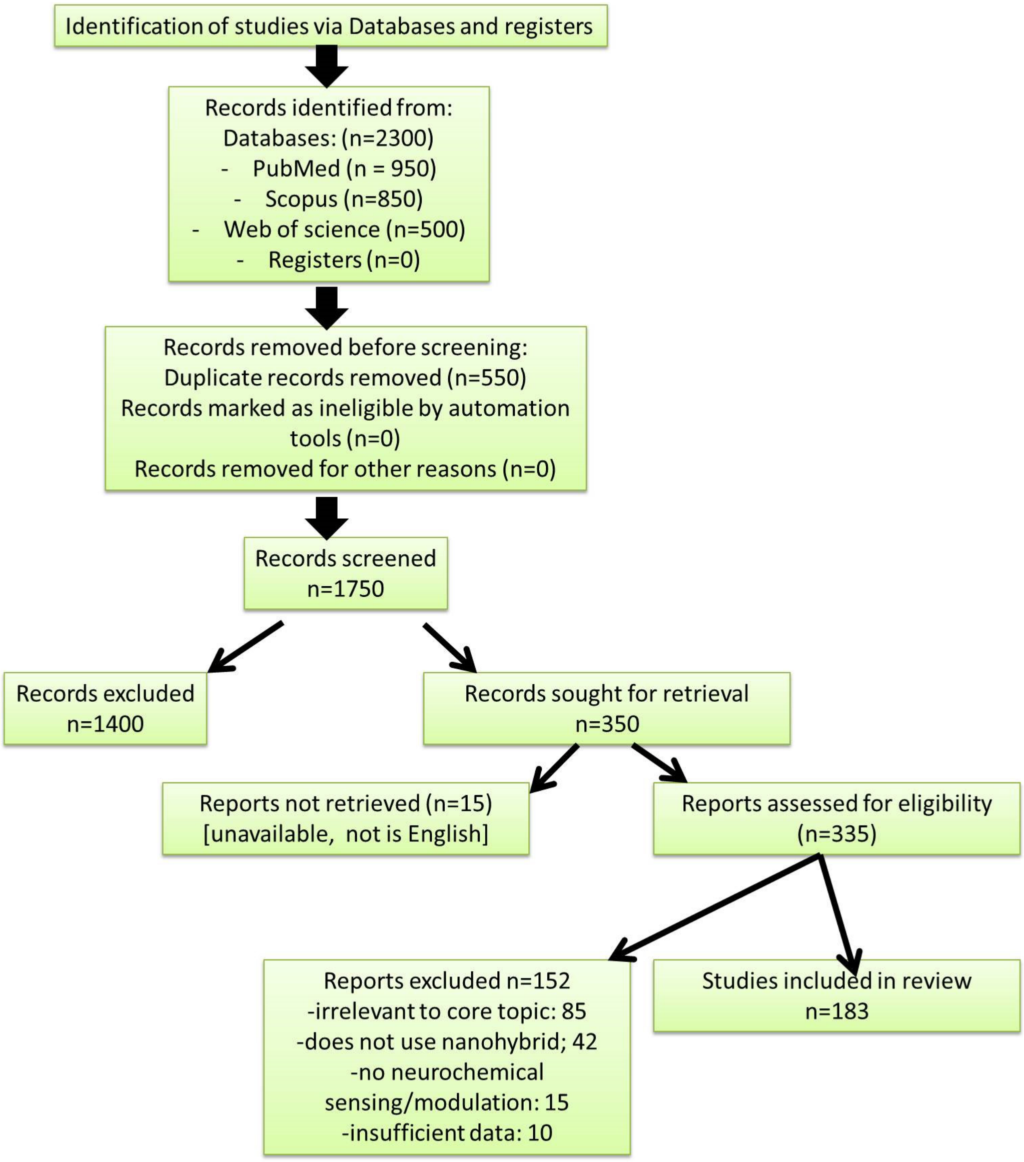

Figure S1. PRISMA 2020 flow diagram illustrating the process of study identification, screening, eligibility assessment, and inclusion in the systematic review.
